# Conducting Polymers in Electrolyte and Separator Engineering for Zn-Ion Hybrid Capacitors

**DOI:** 10.3390/polym18202514

**Published:** 2026-10-14

**Authors:** Thi Thuong Thuong Nguyen, Yichun Su, Cuong Van Le, Hyeonseung Yoon, Ping Liu, Hyeonseok Yoon

**Affiliations:** 1 Department of Polymer Engineering, Graduate School, Chonnam National University, 77 Yongbong-ro, Gwangju 61186, Republic of Korea; 2 School of Petrochemical Engineering, Changzhou University, Changzhou 213164, China; 3 School of Polymer Science and Engineering, Chonnam National University, 77 Yongbong-ro, Gwangju 61186, Republic of Korea; 4 Jiangsu Province Engineering Research Center of Biodegradable Materials, Changzhou University, Changzhou 213164, China

**Keywords:** conducting polymer, electrolyte, separator, hybrid capacitor, energy storage

## Abstract

Conducting polymers (CPs) have emerged as multifunctional components that modulate electrolyte and separator design for Zn-ion hybrid capacitors, with selected Zn-based battery and alkali-ion systems used as mechanistic references rather than direct performance evidence. Their π-conjugated backbones, tunable doping states, and reversible redox chemistry enable coupled ion transport, interfacial charge regulation, and mechanically compliant ion-conducting environments. However, CP integration also requires careful control of electronic leakage, self-discharge, short-circuit risk, and redox-state-dependent swelling, particularly when CP domains are introduced into separator architectures. This review examines how CP-containing electrolytes and separator-related architectures can regulate ion transport and interfacial charge distribution, provided that continuous electronic pathways are suppressed. In aqueous, quasi-solid gel, solid polymer, and redox-active electrolytes, CP-containing domains are examined in terms of ionic conductivity, Zn^2+^ transport, solvation regulation, and polarization mitigation. In separator-related architectures, spatially confined and electronically interrupted CP domains are discussed with emphasis on ion-flux homogenization, dendrite suppression, electron-blocking requirements, and dimensional stability. Comparative analyses separate directly demonstrated Zn-ion hybrid capacitor behavior from mechanisms inferred from related ion-storage systems. Finally, future directions are discussed for quantitative benchmarking, scalable CP processing, and architecture-controlled CP domains for coordinated electrolyte and separator design.

## 1. Introduction

### 1.1. CPs: A Brief Overview

Conducting polymers (CPs) are redox-active conjugated macromolecules that combine electronic charge transport with the processability and mechanical compliance of polymers. Their π-conjugated backbones and reversible doping chemistry enable mixed ionic–electronic conduction, allowing CPs to participate in charge transport and ion compensation in electrochemical systems [1,2]. Since the discovery that chemical doping increased the electrical conductivity of polyacetylene, representative CPs such as polyaniline (PANI), polypyrrole (PPy), and poly(3,4-ethylenedioxythiophene) (PEDOT) have provided model platforms for examining doping-dependent charge transport, redox activity, and environmental stability [3,4,5,6].

From a device perspective, CPs provide a polymeric phase that can couple electron transport, ion exchange, strain accommodation, and retention of interfacial contact. The soft backbones of CPs can reduce mechanical damage caused by the insertion and extraction of ions. The retention of interfacial contact can help maintain electrical connection during repeated electrochemical operation. Owing to these properties, CPs have been studied as electrode materials, interlayers, modifiers of electrolytes, and components of separator-related architectures in electrochemical energy-storage systems [1,2,7]. For Zn-ion hybrid capacitors, the function of CPs depends on the architecture of the cell and on the continuity of CP domains. The electronic conductivity of CPs may assist redistribution of interfacial charge in selected configurations. In contrast, continuous pathways of electron transport across a separator can cause electronic leakage, self-discharge, or short-circuit failure. Therefore, CPs should be evaluated as structure-dependent electrochemical components, rather than as inherently beneficial additives.

At the molecular level, the conductivity of CPs arises from delocalized π-electrons along the conjugated backbone. Oxidation or reduction in the polymer backbone generates polarons and bipolarons. The formation and migration of polarons and bipolarons support electronic charge transport and accompany ion exchange with the surrounding electrolyte [1,2]. The mixed-conduction behavior of CPs links the electronic response of the polymer backbone with ionic compensation in the electrolyte phase. Chemical modification through sulfonation, carboxylation, hydroxylation, or dopant engineering can adjust the affinity of CPs toward electrolytes. The same structural variables also affect interactions between ions and polymer chains, carrier density, redox potential, and dielectric response [8,9,10,11,12,13]. These parameters are relevant to aqueous, quasi-solid, and organic electrolyte environments used in Zn-based hybrid capacitors. Swelling governed by redox state, migration of dopants, softening of polymer domains, and excessive electronic percolation can reduce cycling stability, particularly when CPs are placed near separator or membrane interfaces [14,15,16,17].

The use of CPs in electrolyte and separator engineering requires a design logic different from the use of CPs as electrode materials. In electrolyte matrices, CP-containing domains may contribute to coordination of ions, local charge compensation, redox buffering, and retention of electrolyte wetting. In separator-related architectures, CPs should be considered as spatially confined and electronically interrupted domains rather than continuous electronic films. CP-containing interfacial layers or hybrid membranes may regulate the flux of ions, improve contact between the electrode and separator, and influence the deposition behavior of Zn. The central requirement is to promote ion transport while preventing continuous electronic conduction across the cell. For separator-oriented designs, CP-containing domains require control over spatial distribution, domain connectivity, coating thickness, insulating support, and leakage-current behavior [18,19,20].

The relevance of CPs to Zn-ion hybrid capacitors arises from the coexistence of faradaic and capacitive processes of charge storage. Zn metal anodes often suffer from nonuniform flux of Zn^2+^, dendritic Zn deposition, parasitic reactions, and unstable interfacial resistance. Electrolytes and separators modified with spatially confined CP domains may help regulate the local distribution of Zn^2+^. CP-containing interfacial domains may also retain electrolyte near the electrode surface and moderate charge accumulation near Zn metal. The regulation of Zn^2+^ flux and interfacial charge distribution can support more uniform Zn deposition. Uniform Zn deposition, however, requires that CP-containing domains do not form unintended pathways of electronic conduction between the electrodes. The design of CP-containing electrolytes and separator architectures should therefore balance regulation of ion flux with control of electronic percolation.

Recent advances in vapor-phase polymerization, electrochemical deposition, interfacial polymerization, and self-assembly have improved control over the morphology of CPs, crystallinity of polymer domains, distribution of dopants, and thickness of CP films [8,9,10,11]. In parallel, density functional theory (DFT) and molecular dynamics (MD) simulations have provided molecular-level insight into coordination of ions, interactions between dopants and ions, swelling of polymer domains, and pathways of ion migration in CP-containing matrices [12,13]. Computational and synthetic approaches connect the structure of CPs with selectivity toward ions, kinetics of transport, and stability of interfaces. For electrolyte and separator applications in Zn-ion hybrid capacitors, structure-function relationships must also be interpreted with device-specific requirements. Key requirements include transport of Zn^2+^, suppression of electronic leakage, stability of separators, and resistance to self-discharge. Mechanistic insights from related Zn-ion batteries, alkali-ion systems, and membranes based on non-conducting polymers can guide interpretation. Direct performance evidence obtained from Zn-ion hybrid capacitors should remain separated from evidence inferred from related systems. The distinction between device evidence and mechanistic analogy provides the basis for examining how CP-containing architectures of electrolytes and separators influence performance, stability, and practical feasibility in Zn-ion hybrid capacitors.

### 1.2. Overview of Electrolytes and Separators in Hybrid Capacitors

Hybrid capacitors merge the high energy density of batteries with the high power density of supercapacitors [21,22,23,24]. Their operation depends on the transport of ions through electrolytes and separators, as well as the stability of interfaces formed near electrodes. In Zn-ion hybrid capacitors, electrolyte and separator components must support rapid migration of Zn^2+^ while suppressing parasitic reactions, dendritic Zn deposition, and loss of interfacial contact. CPs can contribute to these functions when introduced as localized electrolyte modifiers, redox-active domains, or spatially confined interfacial components. For separator-related applications, however, CP domains must not form continuous pathways of electronic conduction across the cell. The design of CP-containing electrolytes and separator architectures therefore requires a balance between ion transport, interfacial regulation, and control of electronic leakage.

Throughout this review, a Zn-ion hybrid capacitor denotes an asymmetric cell that couples a battery-type Zn metal (or Zn-storage) negative electrode with a capacitive or pseudocapacitive positive electrode, so that overall performance is bounded simultaneously by faradaic Zn plating/stripping and by rapid interfacial ion adsorption. This device-level definition distinguishes Zn-ion hybrid capacitors from conventional aqueous Zn-ion batteries, in which both electrodes store charge faradaically; from Zn-ion supercapacitors operated with purely capacitive electrodes; and from generic asymmetric supercapacitors that do not involve Zn plating/stripping. This asymmetry is what makes electrolyte and separator design distinctive: the electrolyte must simultaneously support battery-like Zn^2+^ interfacial kinetics at the anode and capacitor-like high-rate ion transport at the cathode, while the separator must suppress dendrite growth without adding resistance that would erode power density.

#### 1.2.1. Electrolyte Classification and Transport Behavior

Electrolytes for hybrid capacitors are generally classified into organic electrolytes, aqueous electrolytes, solid polymer electrolytes, and gel polymer electrolytes. Each electrolyte class provides different advantages and limitations. Organic electrolytes can provide wide electrochemical stability windows, often above 3 V, which can increase the operating voltage of the device. However, organic electrolytes commonly suffer from lower ionic conductivity, volatility, flammability, and safety concerns. Aqueous electrolytes provide high ionic mobility and nonflammability, whereas the narrow electrochemical stability window of water and corrosion-related side reactions restrict voltage operation. Solid polymer electrolytes and gel polymer electrolytes can improve leakage resistance and dimensional stability, although their ionic conductivity often remains lower than that of liquid aqueous electrolytes.

For Zn-ion hybrid capacitors, aqueous electrolytes and quasi-solid or gel polymer electrolytes represent the main electrolyte platforms [25,26,27,28,29]. Organic electrolytes are more appropriately discussed as comparative systems or as media for nonaqueous Zn salt formulations. Mechanistic findings from organic or alkali-ion systems should therefore be separated from direct evidence obtained from Zn-ion hybrid capacitors. The distinction is important because transport of Zn^2+^ is governed by divalent charge, strong hydration, and desolvation barriers that differ from those of monovalent alkali ions.

The electrolyte in a hybrid capacitor must sustain rapid ion migration and stable interfacial reactions under potential gradients generated by faradaic and capacitive charge-storage processes [24]. The electrolyte composition, solvation structure, and interfacial chemistry govern ion mobility, reversibility of charge storage, and cycling stability. Rational electrolyte design therefore requires control over ion coordination strength, dielectric response, water activity, and compatibility with electrode surfaces. CP-containing electrolyte domains may assist ion coordination, local charge compensation, redox buffering, or retention of electrolyte wetting. The beneficial role of CPs should be evaluated together with possible limitations, including redox-state-dependent swelling, dopant migration, increased viscosity, and self-discharge caused by mobile redox species [30,31,32,33,34,35].

#### 1.2.2. Separator Functions and Challenges

The separator is an electronically insulating and ion-permeable component that prevents direct contact between electrodes. In hybrid capacitors, the separator also affects ion-flux distribution, local electric-field uniformity, electrolyte retention, and stability of the electrode–separator interface. The requirements for separators in hybrid capacitors differ from those in conventional batteries or supercapacitors. In batteries, the separator mainly separates electrodes that operate through diffusion-controlled faradaic reactions. In supercapacitors, the separator must permit rapid ion migration for electric-double-layer formation over short diffusion distances. Hybrid capacitors combine faradaic and capacitive processes, requiring separators that sustain high ion flux while maintaining dimensional stability under repeated potential changes.

Several failure modes become important for separators in Zn-ion hybrid capacitors. Nonuniform flux of Zn^2+^ can promote localized current concentration and dendritic Zn deposition. Insufficient electrolyte wetting can increase interfacial resistance and polarization. Chemical instability of the separator can accelerate corrosion, gas evolution, or loss of mechanical strength in aqueous electrolyte environments. Pore-size distribution, tortuosity, surface charge, electrolyte uptake, and adhesion to electrodes therefore influence the cycling stability of Zn-based systems. Separator design should address ion selectivity, electrolyte retention, resistance to pore deformation, and chemical compatibility with both electrodes and electrolyte phases.

CP-containing separator architectures should be discussed with particular caution. A separator must remain electronically insulating, even when CPs are introduced near the separator surface or in an interfacial layer. Accordingly, CPs should be described as spatially confined, electronically discontinuous domains or as components of hybrid separator architectures, rather than as continuous electronically conducting separator films. Localized CP domains may regulate Zn^2+^ flux, alter surface charge, improve electrolyte wetting, and moderate interfacial charge distribution. In contrast, continuous CP networks across a separator can increase leakage current, self-discharge, and short-circuit risk. Therefore, separator-oriented CP designs require control over CP loading, coating thickness, domain connectivity, and electronic percolation.

In aqueous Zn-ion hybrid capacitors, separator design must also consider corrosion and dendrite penetration through porous membranes. Selected alkali-ion hybrid systems can provide mechanistic references for ion-flux regulation because functionalized separator surfaces have been shown to homogenize Li^+^ transport and suppress dendrite growth through tailored surface chemistry and pore architectures [36,37]. The relevance of Li-based separator studies to Zn-ion hybrid capacitors should be presented as mechanistic analogy, rather than direct proof of Zn^2+^-based device behavior. Zn^2+^ hydration, stronger electrostatic interactions, and different plating chemistry require separate validation in Zn-ion hybrid capacitor cells.

#### 1.2.3. Coupled Transport and Interfacial Design

Efficient charge storage in hybrid capacitors requires coordinated ion transport through the electrolyte and separator, coupled with controlled charge transfer at electrode interfaces. The electrolyte and separator should not be regarded as isolated passive components. Instead, their functions are linked through ion migration, solvation and desolvation, electric-field distribution, electrolyte wetting, and interfacial resistance. The relevant design objective is not to create electronic connectivity across the separator. The objective is to maintain fast ion transport while preventing electron transport between electrodes.

CPs may contribute to interfacial design when their redox activity and surface chemistry are spatially controlled. In electrolyte domains, CPs can provide ion-coordination sites, charge-compensation sites, and redox-buffering centers. In separator-related architectures, CP-containing domains may regulate ion flux and improve local wetting near electrode interfaces. The CP phase must remain electronically interrupted in separator applications to avoid leakage current and self-discharge. Mechanical functions should also be described in concrete terms, including strain accommodation, retention of interfacial contact, suppression of pore collapse, and resistance to redox-state-dependent swelling.

These requirements call for a device-specific evaluation framework for CP-containing electrolyte and separator architectures in Zn-ion hybrid capacitors. Key criteria include Zn^2+^ transport, water activity, desolvation behavior, leakage current, self-discharge, dendrite suppression, dimensional stability, and cycling durability. Mechanistic insights from related Zn-ion batteries, Li-ion systems, Na-ion systems, K-ion systems, and membranes based on non-conducting polymers can guide interpretation. Direct claims regarding Zn-ion hybrid capacitors require evidence obtained from Zn-ion hybrid capacitor cells. This distinction strengthens the discussion of CPs as interfacial regulators while addressing the risks associated with electronic conduction in separator environments.

### 1.3. Rationale and Scope of This Review

Although many reviews have examined CPs as electrode materials [2,38,39,40,41], few studies have systematically analyzed how CPs function in electrolytes and separator-related architectures for Zn-ion hybrid capacitors. A focused review of these non-electrode roles is necessary because electrolyte and separator components determine Zn^2+^ transport, water activity, interfacial resistance, self-discharge behavior, and dendrite suppression. In electrolytes, CPs require control over ion coordination, redox-state stability, dopant retention, swelling of polymer domains, and possible redox-shuttle behavior. In separator-related architectures, CP-containing domains require control over electronic insulation, pore accessibility, leakage current, and interruption of electronic percolation. Therefore, CP-containing electrolytes and separator-related architectures should be evaluated through distinct yet connected design criteria.

Herein, we review CPs as modifiers of electrolytes, redox-active polymer domains, localized interfacial layers, and components of hybrid separator architectures. Sections on electrolytes cover CP-containing gel electrolytes, solid polymer electrolytes, and redox-active electrolyte domains that regulate Zn^2+^ transport and interfacial charge compensation. Sections on separators cover insulating separator scaffolds containing spatially confined and electronically interrupted CP domains. Pure CP films are considered only when processing, morphology, or transport principles provide useful guidance for hybrid membrane design. The use of CP-containing domains in separators requires control over electronic insulation because electronically continuous CP phases can increase leakage current, self-discharge, and short-circuit risk.

The dual character of CPs provides the rationale for a balanced review of electrolyte and separator systems. In electrolytes, CP chains, dopants, and redox-active sites can regulate ion solvation, local charge balance, electrolyte wetting, and polarization. Poorly controlled CP domains can also increase viscosity, induce excessive swelling, promote migration of dopants, or accelerate self-discharge. In separator architectures, CP-containing domains can regulate Zn^2+^ flux, local electric-field distribution, and interfacial charge accumulation. Poorly controlled connectivity of CP domains can cause electronic leakage, pore blocking, self-discharge, and short-circuit risk. Critical analysis is therefore required to distinguish useful interfacial functions from failure modes in both electrolyte and separator environments.

Aqueous Zn-ion systems serve as the primary reference because Zn^2+^ transport is governed by divalent charge, hydration structure, desolvation behavior, and Zn plating chemistry. Organic electrolytes, gel-based alkali-ion systems, and membranes based on non-conducting polymers are used selectively as mechanistic references. Related systems help clarify transferable design principles, whereas claims regarding Zn-ion hybrid capacitors require direct evidence from Zn^2+^-based cells. Accordingly, CP-containing electrolyte and separator architectures are evaluated through transport metrics, redox reversibility, swelling behavior, leakage current, self-discharge, electronic insulation, pore retention, dendrite suppression, and cycling stability. Computational modeling, architecture-controlled CP domains, and sustainable polymerization strategies are discussed as future directions for electrolyte and separator engineering.

This review is organized as follows. Section 2 examines CP functions at the electrolyte level, covering gel and solid polymer electrolytes, redox-active electrolyte concepts, mechanistic and transport analyses, and the structure- and doping-state dependence of Zn^2+^ transport in CPs. Section 3 addresses separator-related architectures, including interfacial layers, self-supported membranes, dendrite suppression, durability benchmarks, and design principles. Section 4 develops a device-specific framework connecting electrolyte- and separator-level functions and consolidates the representative systems in table in Section 4.3. Section 5 concludes with prioritized open questions and future directions.

## 2. CPs in Electrolyte Systems

**Figure 1:**
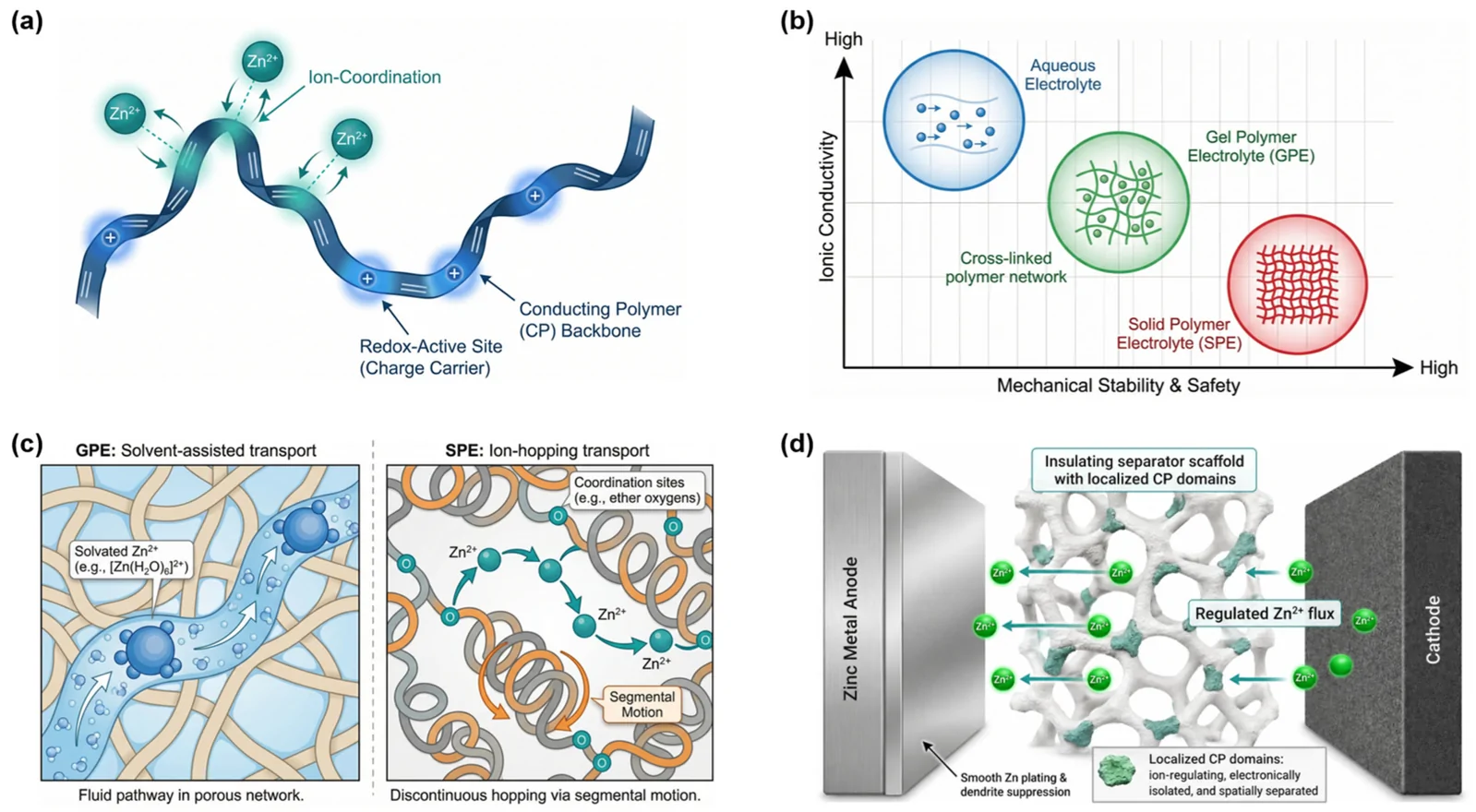
Schematic illustration of CP integration in hybrid capacitor electrolytes. (**a**) Dual functional framework of CPs as ion-coordinating and redox-active components in electrolyte structures. (**b**) Comparative view of aqueous, gel, and solid-state electrolytes, showing the trade-off between ionic conductivity, which is typically higher in aqueous systems, and leakage resistance or dimensional stability, which can be improved in solid-state systems. (**c**) Distinct ion-transport pathways in GPEs and SPEs. GPEs support solvent-assisted transport of solvated Zn^2+^ ions, whereas SPEs operate through ion hopping coupled with segmental motion of polymer chains. (**d**) Interfacial stabilization at the Zn anode, where localized CP-containing interfacial domains regulate ion flux and interfacial charge distribution, thereby suppressing dendritic Zn growth and promoting more uniform metal deposition.

CPs integrated into electrolyte systems provide a route to regulate ion transport, redox activity, and interfacial stability through ion-polymer interactions, redox-mediated charge compensation, and mixed ionic–electronic response (Figure 1a). In contrast to electronically inert polymer matrices that mainly immobilize solvents or salts, CPs contain π-conjugated backbones and redox-active sites that can interact with solvated cations and anions. The interactions between CP chains and electrolyte ions can modify local ion coordination, water or solvent retention, and interfacial polarization. As a result, CP-containing electrolyte domains may support charge compensation and ion migration during high-rate operation in Zn-ion hybrid capacitors. However, the redox activity and electronic conductivity of CPs must be controlled to avoid excessive electronic leakage, redox shuttle behavior, or self-discharge in electrolyte environments.

CPs have been explored in gel polymer electrolytes and solid polymer electrolytes, where their functions depend on the continuity, doping state, and spatial distribution of the CP phase (Figure 1b,c). In gel electrolytes, CP chains or CP-containing domains can provide ion-coordination sites and hydrated pathways for Zn^2+^ migration through electrostatic interactions and redox-assisted charge compensation. The resulting transport behavior should be evaluated using ionic conductivity, Zn^2+^ transference number, electrolyte uptake, and cycling stability, rather than by conductivity enhancement alone. In solid polymer electrolytes, dispersed or confined CP domains can increase the dielectric response of the polymer matrix, weaken ion-pairing interactions, and contribute to segment-assisted ion migration. Electronically continuous CP networks are not always desirable because long-range electronic percolation can increase leakage current and compromise charge retention. Therefore, the design of CP-containing electrolytes requires simultaneous control over ion transport, redox stability, swelling of polymer domains, dopant migration, and compatibility with Zn^2+^-based interfaces (Figure 1d). The following sections examine these mechanisms in gel and solid electrolyte configurations.

### 2.1. Gel Polymer Electrolytes (GPEs)

Gel polymer electrolytes (GPEs) bridge the liquid-like ionic mobility of conventional electrolytes and the dimensional stability of solid-state polymers. Incorporation of CPs can introduce redox-active sites, polar dopants, and ion-coordination motifs into the gel phase. For electrolyte applications, CPs should be considered as redox-active and ion-interacting domains, rather than as continuous electronic networks. The formation of continuous electronic pathways in the electrolyte may increase leakage current, redox shuttle behavior, or self-discharge. Thus, CP-GPE design requires simultaneous control over ion transport, redox stability, swelling of polymer domains, and electronic percolation [42,43,44]. Recent bio-derived aqueous GPEs based on agarose and sodium alginate further illustrate this design space: hydroxyl-rich polysaccharide networks suppressed dendrite and by-product formation on Zn anodes while matching, and at high cathode loading surpassing, a 2 M ZnSO_4_ liquid baseline [45].

In aqueous Zn-ion systems, hydrophilic and heteroatom-containing polymer frameworks with fixed anionic sites can organize water into hydrogen-bonded domains [46,47]. The organized water domains can provide preferential pathways for hydrated Zn^2+^ transport, rather than random diffusion through bulk water. In gels containing sulfonated or carboxylated CP chains, negatively charged groups may anchor water molecules and influence the local coordination environment of Zn^2+^. The resulting hydrogen-bonded domains can assist migration of hydrated Zn^2+^ under an applied electric field. The high water content of the gel can also improve contact between the Zn electrode and electrolyte. Nevertheless, excessive water retention can accelerate hydrogen evolution, corrosion, and swelling of the gel network. Therefore, water activity and electrolyte uptake must be optimized together with ionic conductivity.

**Figure 2:**
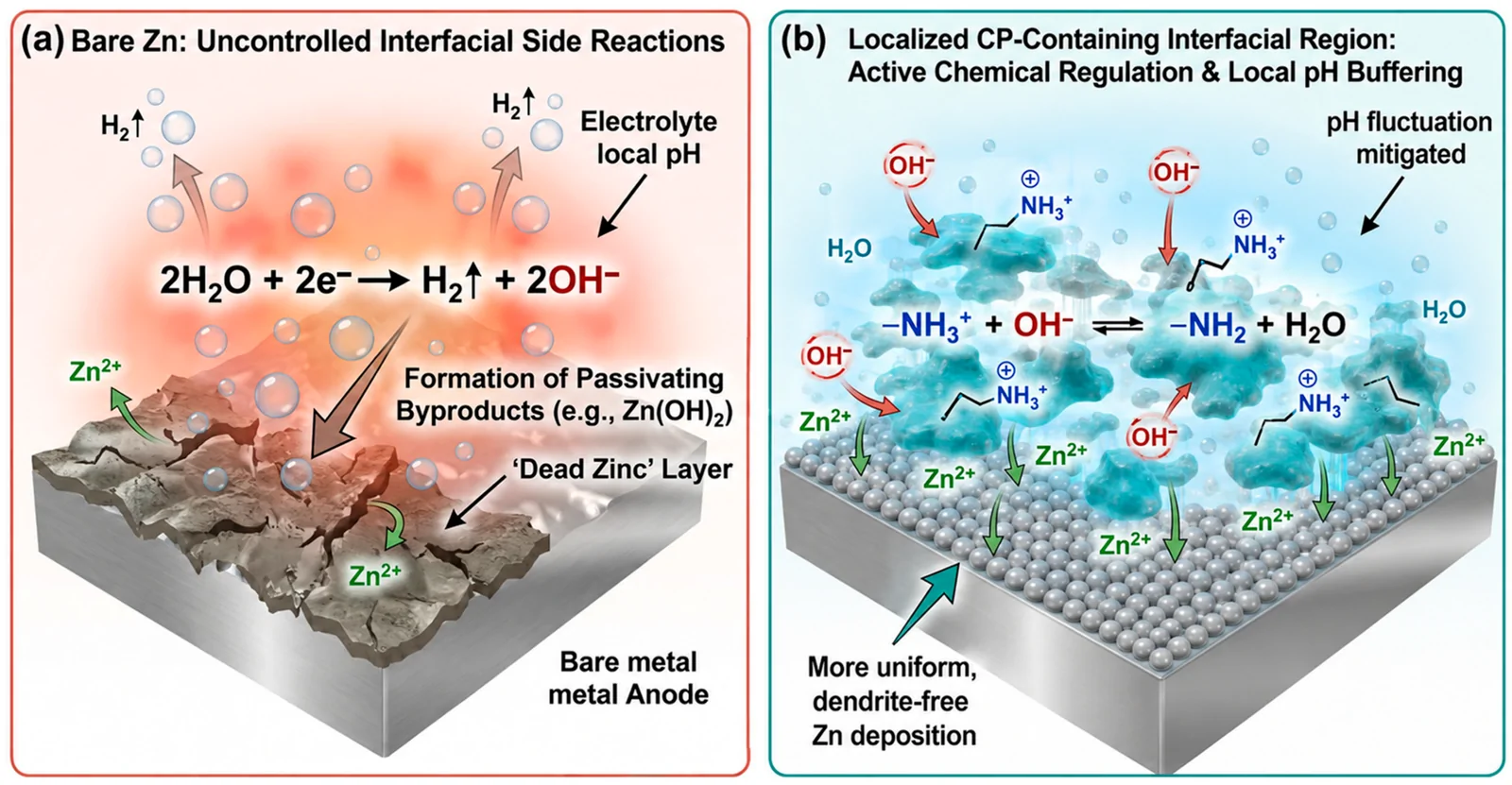
Schematic illustration of the comparative electrochemical environments at the Zn anode interface: (**a**) Bare Zn anode exposed to aqueous electrolyte, where the parasitic hydrogen evolution reaction (HER: 2H_2_O + 2e^−^ → H_2_↑ + 2OH^−^) promotes localized alkalization. The local increase in OH^−^ concentration can induce passivating by-products, such as Zn(OH)_2_ or basic zinc salts, and accelerate nonuniform Zn deposition with dendritic growth. (**b**) Zn anode containing a CP-derived interfacial region. Protonated amine or imine groups in CP chains can buffer locally generated OH^−^ through acid-base neutralization, for example, –NH_3_^+^ + OH^−^ → –NH_2_ + H_2_O. The buffering process can moderate local pH variation, suppress parasitic HER, and promote more uniform Zn deposition with reduced dendritic growth.

Nitrogen-containing conjugated backbones, such as those in PANI [48] or PPy [49], provide redox-active sites capable of participating in transient storage of electrons and protons. Protonated imine, amine, or pyrrolic nitrogen sites may moderate interfacial polarization and local pH variation near Zn electrodes. The moderation of local pH can reduce parasitic hydrogen evolution and improve Coulombic efficiency when the redox process remains confined to the interfacial region. Excessive redox activity, by contrast, may promote redox shuttle behavior or self-discharge if mobile redox species or electronically connected CP domains are present in the gel electrolyte [50,51,52,53]. Figure 2 summarizes the proposed roles of ion-coordination sites, redox-buffering sites, and hydrated Zn^2+^ pathways in CP-containing GPEs.

Recent studies suggest that gels combining fixed anionic groups with redox-active polymer backbones can couple guided Zn^2+^ migration with interfacial charge regulation [53,54,55,56]. Fixed anionic groups can contribute to hydrogen-bonded transport channels for hydrated Zn^2+^, whereas redox-active backbones may buffer charge accumulation near the electrode. The combined regulation of Zn^2+^ flux and interfacial charge distribution has been associated with suppressed hydrogen evolution and improved cycling stability in Zn-ion hybrid capacitors and related Zn-polymer supercapacitors [53,54,55,56]. When the supporting evidence is obtained from Zn-polymer supercapacitors or Zn-ion battery configurations, the mechanism should be treated as related Zn-system evidence rather than direct proof of Zn-ion hybrid capacitor behavior.

Related hydrophilic gels that do not contain conducting polymers also provide useful references for polymer-mediated ion transport. Dueramae et al. [57] demonstrated that carboxymethyl cellulose-based gel electrolytes containing Zn^2+^ produced a dynamic coordination environment. Coordination between Zn^2+^ and carboxylate or hydroxyl groups promoted salt dissociation and reduced crystallinity of the polymer phase. Xiang et al. [46] reported a lean-water hydrogel electrolyte in which sulfonate motifs and hydrogen-bonded water formed lubrication layers and percolated pathways for ion transport. The electrolyte design weakened association of Zn^2+^ and facilitated Zn^2+^ migration under reduced water content. Jin et al. [58] further showed that a carboxyl-rich polyanionic hydrogel formed favorable pathways for Zn^2+^ transport and accelerated desolvation. The improved desolvation behavior enhanced reversibility of Zn plating and stripping by suppressing dendrite growth and side reactions.

A recent review summarized hydrogel electrolyte variables that regulate water activity, solvation structure, and ion-transport pathways at the Zn interface [59]. Reviews on gel polymer electrolytes for alkali-metal systems have also identified segmental-motion-assisted hopping and programmed ion-polymer coordination as important transport motifs [44,60]. These studies provide mechanistic references for CP-GPE design, although monovalent alkali ions and hydrated Zn^2+^ differ in charge density, solvation structure, and desolvation behavior. Therefore, direct translation of alkali-ion gel electrolyte behavior to Zn-ion hybrid capacitors requires separate validation using Zn^2+^-based cells.

Evaluation of CP-GPEs should combine transport metrics with stability metrics. Relevant parameters include ionic conductivity, Zn^2+^ transference number, water activity, electrolyte uptake, swelling ratio, leakage current, self-discharge rate, Coulombic efficiency, and cycling retention. For Zn-ion hybrid capacitors, the value of CP-GPEs lies in controlled Zn^2+^ transport and interfacial charge regulation, provided that redox activity and electronic connectivity do not compromise charge retention.

Two caveats temper these prospects. First, fixed anionic groups exert a competing influence: sulfonate and carboxylate densities high enough to create cation-exchange pathways can also bind divalent Zn^2+^ strongly enough to immobilize it, so that conductivity and transference pass through an optimum rather than increasing monotonically with fixed-charge density. Second, an apparent conductivity enhancement in CP-containing GPEs can arise simply from increased bulk water content rather than from preferential Zn^2+^ channels; distinguishing the two requires Zn^2+^ transference numbers (Bruce–Vincent or Hittorf-type measurements), activation energies from temperature-dependent impedance, and coordination-sensitive spectroscopy (FTIR/Raman shifts in the functional groups, or Zn K-edge X-ray absorption), rather than conductivity alone. Head-to-head comparisons between CP-containing and otherwise identical non-conducting hydrogel electrolytes under the same Zn-ion hybrid capacitor conditions are still missing and are needed to establish whether the CP phase itself, rather than generic hydrogel behavior, provides the benefit.

### 2.2. Solid Polymer Electrolytes (SPEs)

**Figure 3:**
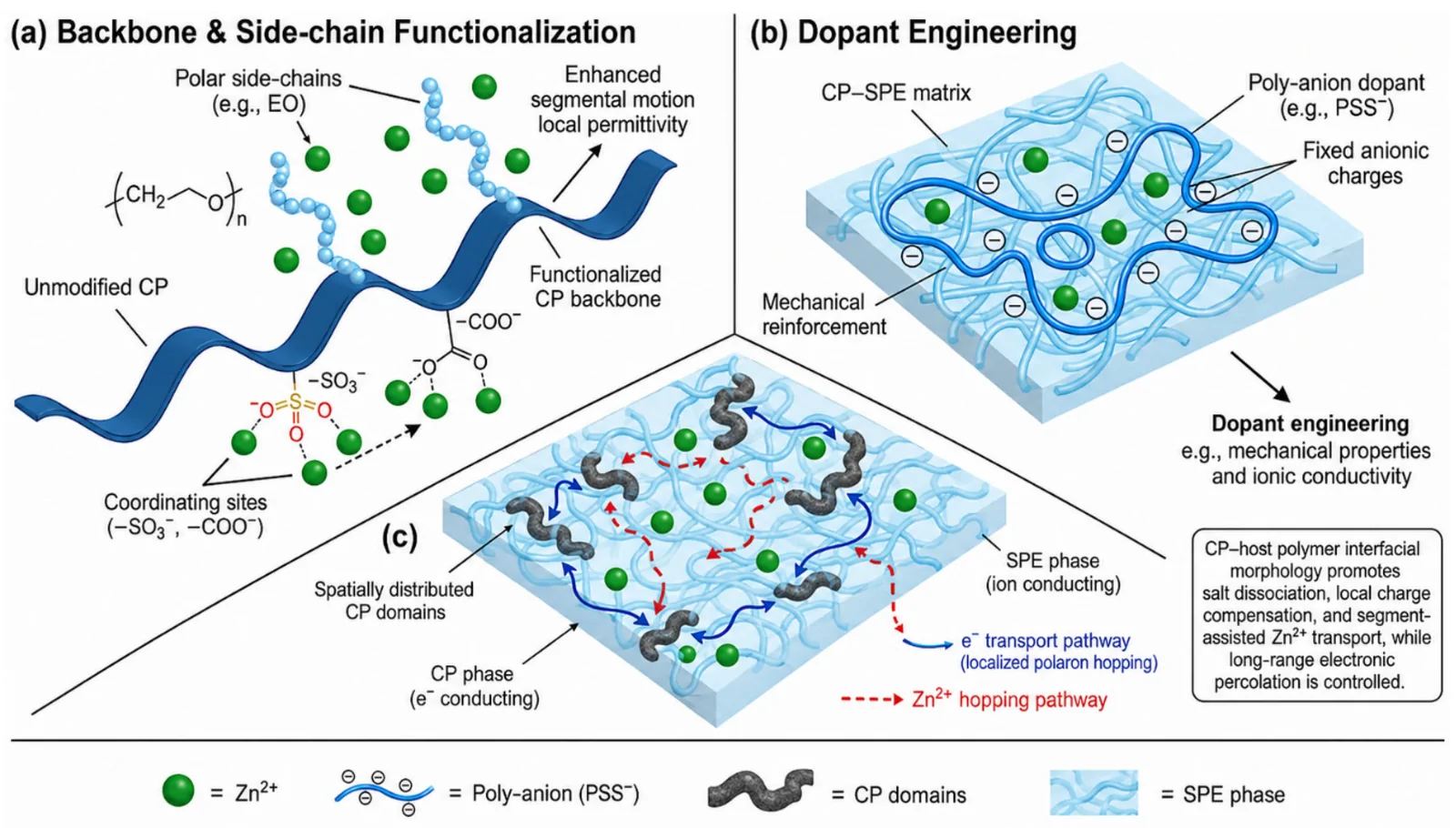
Key structural design strategies for solid polymer electrolytes containing CP domains (CP–SPEs). (**a**) Backbone functionalization, where polar side chains (e.g., –CH_2_CH_2_O–, –SO_3_^−^, –COO^−^) are introduced to create Zn^2+^ coordination sites and tune material properties (dielectric response ε, glass transition temperature T_g_, crystallinity). (**b**) Dopant engineering, using additives such as ionic liquids or polyanions to control mechanical properties and modulate both electronic (σ_elec_) and ionic (σ_ion_) conductivity. (**c**) CP-host polymer interfacial morphology, where spatially distributed CP domains contact the Zn^2+^-hopping polymer phase. The CP-host polymer interface can promote salt dissociation, local charge compensation, and segment-assisted Zn^2+^ transport, whereas long-range electronic percolation should be controlled to avoid leakage current and self-discharge.

SPEs for hybrid capacitors conduct ions mainly through coordination with polar polymer segments and segmental motion of the host matrix [61]. In contrast to gel electrolytes, SPEs contain little or no free liquid phase. The absence of free solvent can improve dimensional stability and reduce electrolyte leakage. The same solid-state structure often limits ion mobility at ambient temperature because cation migration depends on the mobility of polymer segments and the strength of ion-polymer coordination. Figure 3 summarizes structural design strategies for CP-containing solid polymer electrolytes (CP-SPEs).

Functionalization of polymer backbones introduces polar side chains, including ethylene oxide, sulfonate, carboxylate, or phosphonate groups (Figure 3a). The polar groups can increase local permittivity, provide coordination sites for Zn^2+^, and regulate the glass-transition temperature and crystallinity of the polymer phase. Dopant engineering uses ionic-liquid-like additives, polyanions, or fixed ionic groups to modulate salt dissociation, mechanical response, and the continuity of ion-conducting domains (Figure 3b). Architectures combining CP domains with Zn^2+^-hopping host polymers can create nanoscale CP-polymer interfaces (Figure 3c) [61,62]. The role of these interfaces is to promote salt dissociation and local charge compensation. The CP phase should remain electronically confined or discontinuous when charge retention is required, because long-range electronic percolation can increase leakage current and self-discharge.

Incorporation of CPs into SPE architectures may mitigate interfacial resistance by introducing polar and redox-active domains near the electrode–electrolyte interface. The CP domains can improve local wetting, provide additional cation-coordination sites, and regulate charge accumulation at the interface. Early solid-state Li cells employing PANI with a polymer electrolyte showed that contact between PANI and the electrolyte affected polarization and interfacial impedance [63]. Impedance analysis further indicated that interfaces involving Li, PANI, and modified poly(ethylene oxide) (PEO) electrolytes exhibited lower interfacial resistance after interface engineering [63]. More recently, a PEDOT:poly(styrene sulfonate) (PEDOT:PSS)-PEO hybrid layer was reported as a separator-related coating or interfacial layer in Li batteries. The layer improved interfacial contact and reduced polarization relative to CP-free analogs [64].

The Li-based studies provide useful mechanistic references for CP-SPE design. Segmental-motion-assisted ion hopping in polar PEO-based matrices, salt dissociation at CP-polymer interfaces, and reduced charge-transfer resistance at conformal interfacial layers can inform the design of Zn^2+^-conducting SPEs [61,62,63,64,65]. However, Li^+^ systems should not be treated as direct evidence for Zn-ion hybrid capacitors. Zn^2+^ migration involves divalent charge, stronger electrostatic interactions, and a different solvation or desolvation environment. Therefore, Zn^2+^-conducting CP-SPEs require validation through Zn-based cells and transport metrics, including ionic conductivity, Zn^2+^ transference number, leakage current, self-discharge rate, and cycling retention.

In CP-SPE architectures, interfaces between CP domains and host polymers such as PEO, poly(vinylidene fluoride) (PVDF), or poly(methyl methacrylate) (PMMA) can form nanoscale regions that affect salt dissociation and cation hopping [61,62,64,65]. The interfacial effect depends on the polarity of functional groups, distribution of dopants, amorphous fraction of the host polymer, and connectivity of the CP phase. Electronic connectivity should be controlled separately from ion connectivity. Ionic pathways are required for Zn^2+^ migration, whereas unintended electronic pathways can compromise charge retention.

**Figure 4:**
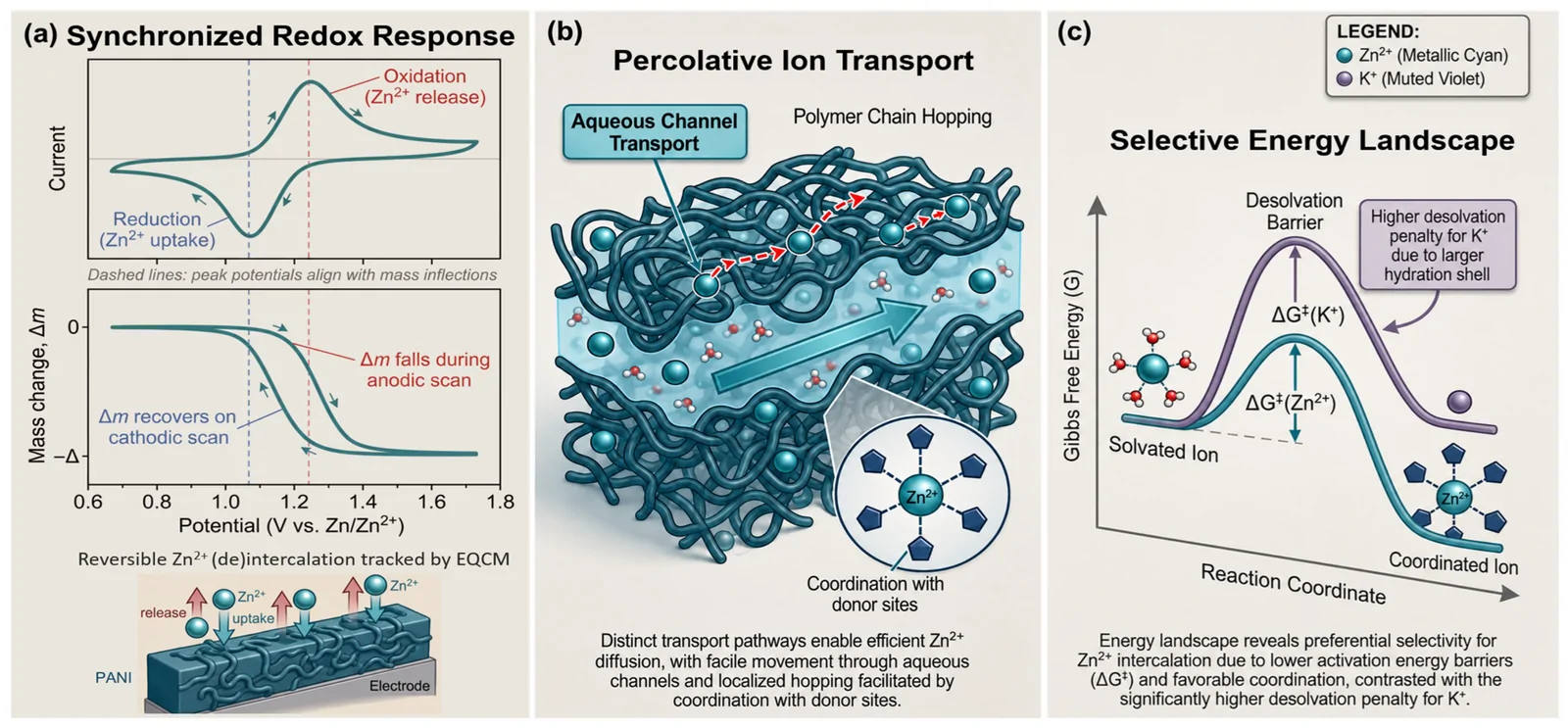
Ion transport and interfacial regulation in CP-containing interphases. (**a**) Coupled electrochemical and mass response of a PANI-coated electrode. Correlation between cyclic voltammetry (CV) and electrochemical quartz crystal microbalance (EQCM) signals indicates that charge storage involves backbone redox reactions coupled with ion exchange. The current and mass-change traces are drawn on a common potential axis so that redox peaks and mass inflections can be compared directly. (**b**) Schematic illustration of percolative ion-transport pathways in a CP-containing interphase. Ions can migrate through hydrated nanochannels or hop between coordination sites, such as nitrogen-containing sites, along polymer chains. (**c**) Gibbs free-energy landscape of ion permeation across the CP-containing interphase. The interphase lowers the desolvation activation barrier (ΔG^‡^) for Zn^2+^ and produces ion-specific permeation barriers, indicating that selective transport depends on hydration, desolvation, and coordination with CP functional sites.

Figure 4 provides a mechanistic framework linking CP-containing interphase variables to measurable transport and interfacial outputs. Cyclic voltammetry-electrochemical quartz crystal microbalance (CV-EQCM) analysis of a PANI-coated electrode supported redox-coupled uptake and release of Zn^2+^ at the CP interphase (Figure 4a) [66]. The result indicates that the CP domain participated in charge regulation at the electrode interface, rather than acting only as a wetting layer. The CP interphase also rationalizes possible ion-transport modes, including migration through hydrated nanochannels and hopping between coordination sites along polymer chains (Figure 4b) [13]. The relative contribution of each transport mode depends on the polarity of functional groups, dopant distribution, and amorphous character of the polymer phase. The free-energy landscape in Figure 4c indicates that a CP-containing interphase can lower the desolvation activation barrier (ΔG^‡^) for Zn^2+^ [67]. The same interphase may impose a larger kinetic penalty on cations with larger hydration shells. The energy-barrier difference provides a physical basis for ion selectivity and reduced charge-transfer resistance at the electrode–electrolyte boundary. The interpretation should remain limited to the specific interphase and cell configuration tested. Broader claims for Zn-ion hybrid capacitors require direct measurements in full hybrid capacitor cells.

Solid and quasi-solid Zn^2+^ polymer electrolytes that do not contain CPs also provide important design references for CP-SPEs. Yao et al. [68] reviewed solid-state electrolytes for Zn batteries and emphasized that polar functional groups and high dielectric-constant matrices promoted salt solvation and more homogeneous Zn^2+^ transport, while crosslinked networks balanced mechanical stability with ionic conduction and mitigated dendrite growth. Yang et al. [69] designed a quasi-solid polymer electrolyte by crosslinking sodium polyacrylate with lithium magnesium silicate and cellulose nanofiber. The negatively charged ion pathways modulated Zn^2+^ desolvation, facilitated ion transport, improved Zn plating-stripping reversibility, and suppressed parasitic reactions. In a different solid-electrolyte strategy, Yan et al. reported a polymer-in-salt Zn^2+^-conductive solid electrolyte prepared by a fast phase-transition route for stable Zn batteries [70].

CP-SPEs are more accurately described as architecture-controlled solid electrolyte systems than as electronically conducting polymer networks. The main design objective is to increase Zn^2+^ mobility and interfacial compatibility while preserving charge retention. Effective CP-SPE design therefore requires polar coordination sites, controlled amorphous domains, mechanically stable host matrices, and electronically interrupted CP domains. Quantitative evaluation should include ionic conductivity, Zn^2+^ transference number, interfacial resistance, leakage current, self-discharge behavior, dimensional stability, and cycling durability.

### 2.3. Redox-Active Electrolytes and Polymer Additives

Beyond structural roles in gel and solid polymer electrolytes, redox-active polymers and CP-derived species can introduce charge-compensation sites into the electrolyte phase. Potential configurations include immobilized redox segments, colloidal CP domains, strongly swollen polymer networks, and soluble CP oligomers. The distinction among immobilized, dispersed, and soluble redox species is important because their effects on charge storage and self-discharge can differ substantially. Immobilized or spatially confined redox sites may buffer local potential gradients and regulate interfacial charge accumulation. In contrast, mobile redox species can promote redox shuttle reactions, leakage current, and self-discharge when their diffusion is not controlled [71].

**Figure 5:**
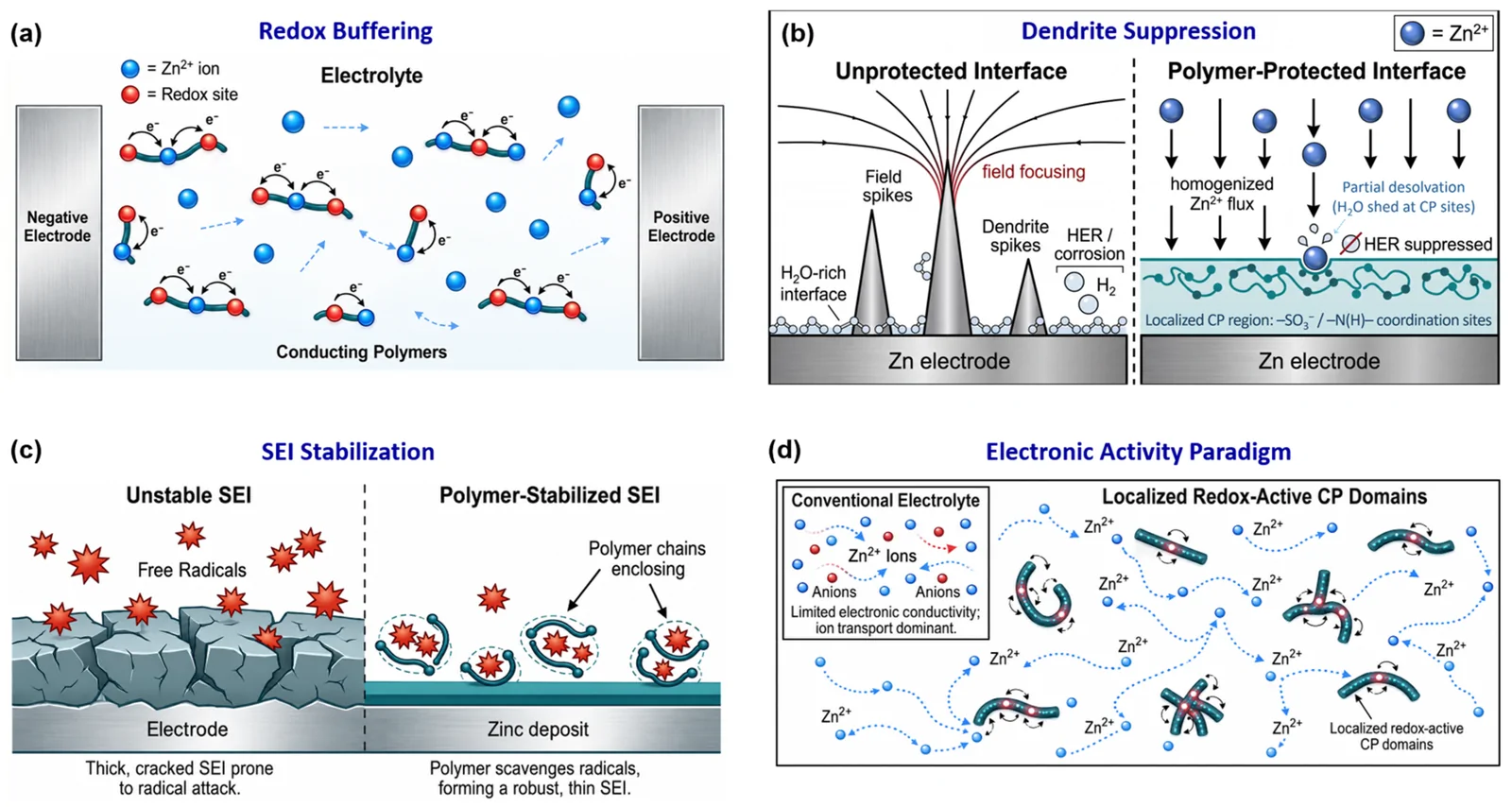
Schematic illustration of the multifunctional roles of CP-derived redox-active components in Zn-ion hybrid capacitor electrolytes. (**a**) Redox mediation and potential buffering: Localized CP redox sites facilitate interfacial charge buffering and short-range electron hopping, thereby smoothing local potential gradients without forming long-range electronic pathways. (**b**) Regulation of Zn deposition: A comparison between nonuniform Zn deposition and CP-regulated interfacial chemistry illustrates how CP-derived or CP-containing species can redistribute local Zn^2+^ flux and interfacial charge accumulation, thereby mitigating tip-enhanced deposition and promoting smoother Zn plating. The panel contrasts interfacial water enrichment at the unprotected Zn surface with partial desolvation, Zn^2+^ coordination at CP functional sites, and suppressed hydrogen evolution at the protected interface. (**c**) Solvent and interphase stabilization: Redox-active or radical-scavenging polymer species can suppress reactive intermediates generated during cycling, reducing solvent decomposition and supporting the formation of a more uniform interphase or passivation layer relative to unstable interphases formed in conventional electrolytes. (**d**) Emerging concept of redox-active electrolytes with controlled electronic coupling: Localized CP-derived redox domains can introduce electrolyte-phase charge buffering while preserving ion-dominated transport. The design requires controlled redox-site distribution and limited long-range electronic connectivity to avoid leakage current, self-discharge, and uncontrolled shuttle reactions.

Accordingly, redox-active electrolytes should not be described as electrolytes that require long-range electronic conduction between electrodes. The intended function is localized redox buffering coupled with ion transport, rather than continuous electron transport through the electrolyte. For Zn-ion hybrid capacitors, CP-derived redox units may provide additional faradaic buffering beyond electrode surfaces. However, direct demonstrations of soluble or dispersed CP-derived redox additives in Zn-ion hybrid capacitor electrolytes remain limited. Small-molecule redox additives, radical polymer gels, and redox-active hydrogel electrolytes therefore provide mechanistic references rather than direct device evidence [71,72,73]. Figure 5 summarizes the possible roles of CP-derived electrolyte additives, including redox mediation, potential buffering, regulation of Zn deposition, suppression of solvent decomposition, and stabilization of interfacial chemistry.

#### 2.3.1. Redox Mediation and Potential Buffering

Redox mediators dissolved in electrolytes or immobilized in polymer matrices can provide distributed faradaic charge storage. The additional redox contribution can increase apparent capacitance when electron exchange remains reversible and spatially controlled [71]. However, redox shuttle reactions can accelerate self-discharge and reduce cycling stability. Therefore, redox mediation requires control over mediator diffusion, redox potential, chemical stability, and compatibility with electrode interfaces.

Vlad et al. [72] demonstrated that organic radical polymer gel materials supported fast bulk-redox charge storage in carbon-redox polymer-gel hybrid supercapacitors. The redox process occurred with surface double-layer ion exchange at carbon electrodes, enabling high-rate discharge in a carbon-redox polymer-gel configuration. Xun and co-workers [73] reported an ultrathin redox-active hydrogel electrolyte containing the redox-active ionic liquid [ViEtIm][Br] in a poly(vinyl alcohol) (PVA) and carboxymethyl chitosan network. The redox reaction of the ionic liquid provided a pseudocapacitive contribution to flexible supercapacitors. A review article on aqueous redox additives summarized that redox species in aqueous electrolytes can enhance charge storage via reversible redox transformations occurring at or near porous carbon, while highlighting redox-shuttling-induced self-discharge as a key limitation [71]. Note that the above examples mainly involve non-CP mediators. Therefore, their relevance to CP-derived electrolyte additives should be framed as mechanistic analogy. Reversible electron exchange in an electrolyte-swollen polymer phase may guide the design of soluble CP derivatives or short CP oligomers. Nevertheless, the use of CP-derived redox additives in Zn-ion hybrid capacitors would require direct validation of redox reversibility, mediator retention, leakage current, self-discharge behavior, and long-term chemical stability.

#### 2.3.2. Dendrite Suppression, Solvent Stability, and Interfacial Regulation

In aqueous Zn-based systems, mechanistic evidence for CP-mediated interfacial regulation has mainly been obtained from CP phases anchored at or near the Zn metal–electrolyte boundary. The evidence does not necessarily represent freely dissolved CP additives in bulk electrolytes. Zhang et al. [56] reported an in situ-formed Zn^2+^-crosslinked PEDOT:PSS interphase on Zn metal. The interphase homogenized the interfacial electric field and Zn^2+^ flux, suppressed side reactions, and enabled dendrite-free Zn deposition with extended cycling stability. Ma et al. [48] demonstrated that phytic-acid doping of PANI together with zinc phytate as an electrolyte additive mitigated water attack on imine sites and improved the stability of Zn-PANI chemistry, while also indicating suppression of parasitic reactions including hydrogen evolution. A recent review further summarized that CP coatings or interlayers on Zn anodes can suppress dendrite nucleation and promote homogeneous ion flux across Zn-based hybrid devices [2].

The extension of anchored CP interphases to soluble CP fragments or sulfonated CP derivatives in Zn electrolytes should be presented as a design hypothesis. Spatial anchoring of CP redox sites, retention of redox species, and resistance to side reactions can determine mediator lifetime. Soluble CP-derived species may improve redox buffering only when uncontrolled diffusion, shuttle reactions, and electronic leakage are suppressed. For the broader concept of redox mediation in metal batteries, reviews of Li-O_2_ systems provide established frameworks for evaluating how soluble redox mediators affect parasitic chemistry and interfacial stability [74]. A review of aqueous redox additives also provides relevant criteria for discussing benefits and limitations of mediator-assisted charge storage in aqueous electrolytes [71].

#### 2.3.3. Emerging Paradigm: Redox-Active Electrolytes with Controlled Electronic Coupling

The studies discussed in Section 2.3.1 and Section 2.3.2 suggest an emerging design paradigm for redox-active electrolytes. Electrolyte phases can contain redox-active components while retaining their primary role as ion-conducting media. The key requirement is controlled electronic coupling. Redox-active species should provide local charge buffering or pseudocapacitive contribution without forming continuous electronic pathways between electrodes. Therefore, redox-active electrolytes require evaluation of charge retention, leakage current, self-discharge behavior, and chemical stability, in addition to any increase in apparent capacitance [71,72,73,74,75].

Redox-active polymer gels and hydrogel electrolytes show that electrolyte-rich polymer phases can contribute faradaic or pseudocapacitive charge storage while sustaining ion transport [72,73,75]. CP-mediated Zn interphases further indicate that redox-active polymer domains can regulate interfacial charge distribution and Zn^2+^ flux when redox sites are spatially anchored near the metal–electrolyte boundary [48,56]. These findings provide a conceptual basis for extending redox-active electrolyte design toward CP-derived species in Zn-based hybrid capacitors.

Building on these analogs, CP-derived redox additives could be explored as an extension for Zn-based hybrid capacitors. Candidate structures include short sulfonated PANI chains, low-molecular-weight PEDOT, and other solvent-compatible CP derivatives. Conjugated backbones and heteroatom-containing redox centers may enable coupled electron-ion mediation and local potential buffering in the electrolyte phase. Future studies should determine whether these species can remain immobilized or spatially confined while preserving reversible redox response under Zn^2+^-based operating conditions.

For redox-active electrolyte concepts, the boundary between beneficial buffering and harmful shuttling must be established experimentally rather than assumed. The two regimes can be discriminated by scan-rate-dependent voltammetry (a shuttle current persists at low rates and scales with mediator diffusion), by float-current and self-discharge tests at fixed states of charge, and by comparing the Coulombic efficiency of Zn plating/stripping with and without the additive; any additive-induced efficiency loss or steady parasitic current indicates shuttle-dominated behavior. Because soluble CP derivatives are electronically active, their beneficial concentration window is expected to be narrow and system-specific, bounded from above by the onset of measurable leakage current and self-discharge; no such window has yet been established for Zn-ion hybrid capacitors, and the concepts summarized in this section should accordingly be regarded as proposed rather than demonstrated (Table in Section 4.3).

### 2.4. Mechanistic Insights and Transport Coupling

In CP-containing electrolyte systems, electrochemical doping links the redox state of the conjugated backbone with ionic compensation from the electrolyte [76,77]. The transport of electrons along CP chains and the migration of ions in electrolyte-swollen polymer domains therefore occur through coupled processes. Ionic motion preserves local electroneutrality and affects the rate of redox-state conversion in the CP phase. For electrolyte and separator-related applications, however, ion-electron coupling should be interpreted as local charge compensation rather than long-range electronic conduction across the cell.

**Figure 6:**
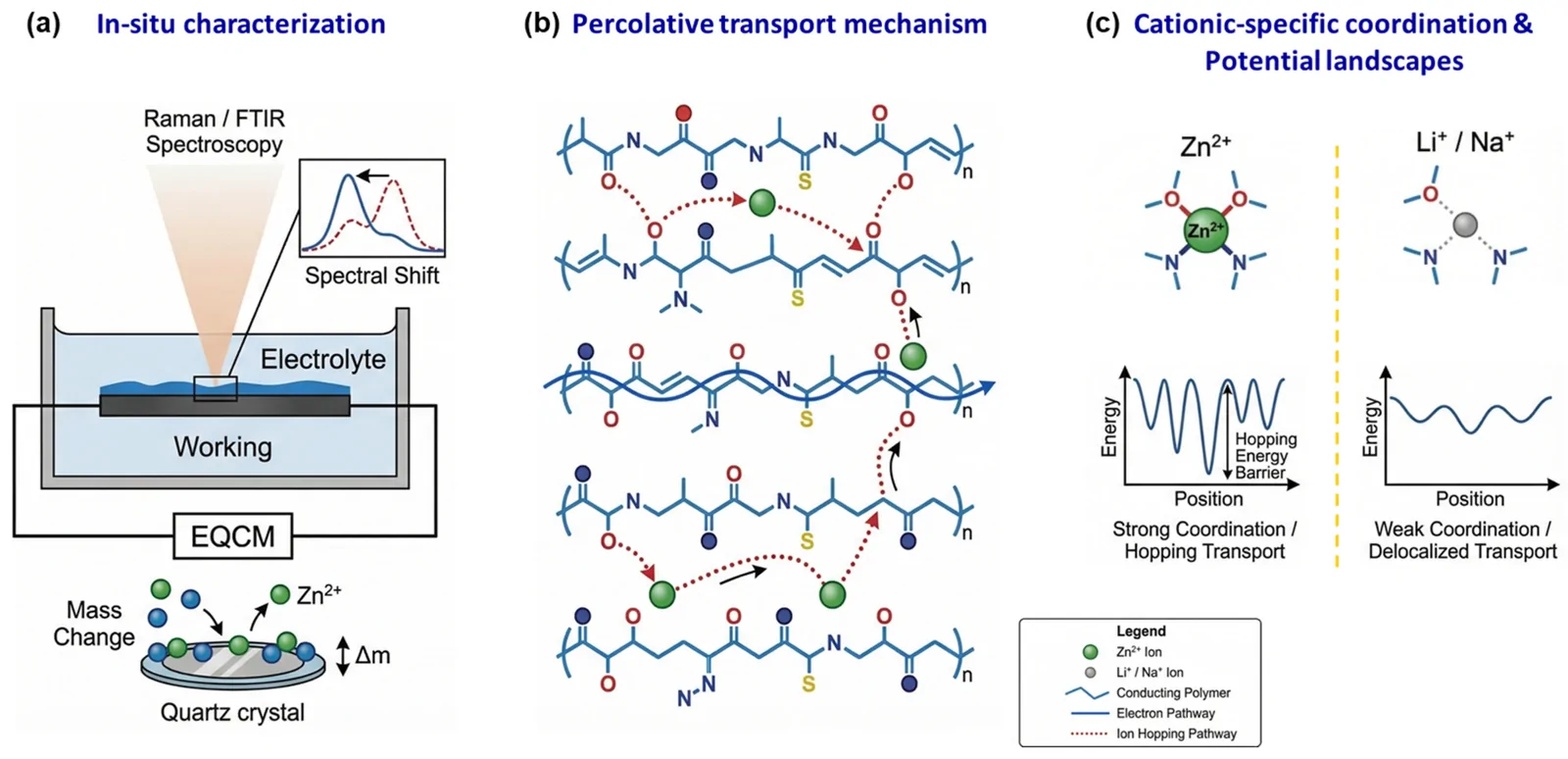
Mechanistic insights into ion transport and localized electronic response in CP-containing electrolytes. CP backbones provide redox-active domains for short-range polaron delocalization, while free volume, solvated regions, and coordination sites support ion migration. (**a**) In situ spectroscopic and gravimetric fingerprints. Raman or Fourier transform infrared (FTIR) spectroscopy tracks potential-dependent changes in CP vibrational bands, such as C–N^+^ and C=C modes, while EQCM monitors coupled uptake and release of ions and solvent during redox cycling. (**b**) Percolative ion transport. CP-containing microstructures provide dynamic ion-migration pathways through transient coordination sites associated with heteroatoms or dopant groups. Local electronic response occurs inside CP-rich domains, whereas long-range electronic percolation should be controlled to prevent leakage current and self-discharge. (**c**) Cation-specific coordination and potential landscapes. Zn^2+^ is shown as a reference cation because donor chemistry, hydration structure, and electrolyte composition can produce stronger coordination, deeper local potential wells, and higher desolvation or hopping barriers than those of monovalent alkali ions. Li^+^ and Na^+^ serve as mechanistic benchmarks for comparing the effects of ion valence, solvation, and coordination strength.

The strength of coupling depends on polymer morphology, dopant chemistry, and the distribution of polar functional groups [78]. Coordinating sites and dopant anions define local solvation pockets and determine the connectivity of ion-transport pathways. Zn^2+^ generally exhibits stronger ion-polymer interactions and slower site-exchange kinetics than monovalent cations [79]. The transport of Zn^2+^ is therefore sensitive to donor identity, hydration structure, polymer relaxation, and the spacing of coordination sites. Figure 6 summarizes the mechanistic framework for coupled transport. The framework links spectroscopic and gravimetric fingerprints (Figure 6a), connectivity-governed ion transport (Figure 6b), and cation-specific coordination landscapes benchmarked against alkali cations (Figure 6c).

#### 2.4.1. In Situ Spectroscopic and Gravimetric Evidence

In situ vibrational spectroscopy and electrogravimetric analysis provide complementary evidence for ion-coupled redox transport in CPs. Vibrational spectroscopy tracks changes in the chemical structure of the conjugated backbone, whereas electrogravimetric analysis quantifies mass changes associated with ion and solvent exchange. The combination of these methods is therefore useful for separating redox conversion of the polymer backbone from charge compensation by mobile ionic species.

In situ FTIR studies of PANI in acidic media tracked potential-dependent changes in quinoid and benzenoid motifs [80]. The evolution of these bands provided spectroscopic markers for redox conversion of the PANI backbone. EQCM measurements further quantified the accompanying mass response during PANI oxidation [81]. The mass change revealed a transition from anion insertion to proton expulsion during the leucoemeraldine-to-emeraldine conversion, depending on acid concentration. The coupled spectral and mass responses established ion-exchange stoichiometry as a key component of PANI redox chemistry [80,81].

EQCM-D analysis of PANI electrodes in aqueous Na^+^-containing electrolytes extended the ion-exchange picture by resolving mixed anion and proton fluxes together with water uptake [82]. The measured fluxes depended on electrolyte pH and anion identity, indicating that CP redox behavior is controlled by both backbone chemistry and electrolyte composition. A Zn-specific EQCM-Raman study [66] in aqueous Zn(TfO)_2_ electrolyte further correlated potential-dependent Raman signatures with reversible mass changes. Raman bands (e.g., C–N^+^ and C=C vibrations) were linked to backbone redox evolution, whereas the EQCM response identified the ionic species involved in charge compensation. This study supported a Zn-storage mechanism in which TfO^−^ insertion dominated charge compensation, while Zn^2+^ participation appeared under selected electrochemical conditions.

Consistent with the need for careful Raman assignment, Raman analysis of PEDOT [83] further showed that the C=C symmetric stretching region responds sensitively to the electrochemical and ionic environment. The PEDOT case is important because shifts in the C=C band can originate from several coupled factors, including changes in redox state, chain conformation, chain packing, and microstructural organization. Reliable identification of Zn^2+^ participation in CP-based interphases therefore requires Raman assignments to be supported by gravimetric or electrochemical measurements.

#### 2.4.2. Connectivity-Governed Ion Transport in CP-Based Electrolytes

MD simulations and theoretical analyses of polymer electrolytes provide a microscopic framework for ion motion in CP-rich environments. Simulations of ether- and carbonyl-containing polymer electrolytes showed that cations migrated via hopping among transient coordination sites formed by donor atoms and anion-associated groups [84,85]. The resulting ion transport often follows sub-diffusive dynamics on a fluctuating lattice of favorable solvation environments, rather than Fickian diffusion in a uniform medium.

A percolation-based view further indicates that macroscopic conductivity in multiphase polymer electrolytes can depend on a critical connectivity threshold of solvation sites [86]. The threshold links mesoscale morphology with the availability of low-barrier ion pathways. Many canonical simulations have focused on non-conjugated hosts and Li-based salts. Nevertheless, the connectivity-based transport picture provides a useful mechanistic reference for CP-containing electrolytes and CP-modified interphases.

In CP-containing domains, heteroatom sites can generate heterogeneous coordination environments for Zn^2+^. Relevant donor sites include nitrogen atoms in PANI or PPy and sulfur or oxygen atoms in thiophene-based CPs or polar side chains. Dopant anions and hydrated Zn^2+^ further modify the local free-energy landscape for ion migration. Electrochemical doping can change ion uptake, water uptake, polymer packing, and swelling of CP domains [77,87]. As a result, connectivity of ion pathways and polaron transport can change with redox state.

For Zn-ion hybrid capacitors, CP domains should therefore be viewed as local transport-regulating regions. Heteroatom coordination, dopant distribution, and redox-state-dependent swelling can set the density and connectivity of Zn^2+^ hopping pathways. Electronic connectivity must be controlled separately from ionic connectivity. Local electronic response may support redox compensation at an interphase, whereas extended electronic pathways through electrolyte or separator regions can increase leakage current and self-discharge. Alkali-ion polymer electrolytes remain useful mechanistic analogs for pathway-connectivity arguments. Zn^2+^ systems require direct validation because stronger coordination and slower site exchange increase sensitivity to local solvation topology [2].

#### 2.4.3. Cation-Specific Coordination and Potential Landscapes

Ion-coupled transport in CP-containing electrolytes depends strongly on cation identity. For Zn-ion hybrid capacitors, the central issue is how Zn^2+^ interacts with donor motifs in the polymer phase. Zn^2+^ has high charge density and often forms strong, multidentate coordination with donor-rich polymer segments [79]. Strong coordination can deepen local free-energy minima and promote activated site-to-site hopping. The transport behavior differs from shallow-landscape diffusion that is more typical for weakly bound ions.

Single-ion-type polyanionic polymer environments designed for aqueous Zn batteries provide relevant Zn^2+^ analogs [88]. In those systems, Zn^2+^ migration has been described as hopping between anion-tethered coordination environments. Local relaxation of the polymer phase assists the hopping process. The same coordination-pocket concept can inform the design of CP-containing Zn^2+^ electrolytes. Nitrogen-rich backbones, oxygen-containing side chains, and dopant anions can define discrete Zn^2+^ coordination pockets. Zn^2+^ mobility is then governed by pocket spacing, donor identity, hydration state, and connectivity of low-barrier pathways.

Comparisons with monovalent cations should remain qualitative unless direct measurements are available in matched cell configurations. Monovalent ions generally show weaker effective binding and broader coordination distributions in polymer electrolytes [89]. The weaker binding is often associated with shallower effective energy landscapes. In contrast, Zn^2+^ transport depends more strongly on coordination strength, desolvation barrier, and local polymer relaxation. Therefore, alkali-ion data can support mechanistic interpretation, whereas claims regarding Zn-ion hybrid capacitors require Zn^2+^-based transport measurements and device-level validation.

#### 2.4.4. Structure-, Doping-State-, and Dopant-Dependent Transport

Although PANI, PPy, and PEDOT are often discussed collectively, their Zn^2+^-relevant chemistry differs substantially. PANI offers protonatable amine/imine nitrogen sites whose Zn^2+^ affinity and hydrophilicity are strongly pH- and doping-state-dependent; PPy provides softer N–H donor sites and typically more disordered, hydrated films; PEDOT contributes thiophene sulfur with only weak direct Zn^2+^ coordination, so that in PEDOT:PSS systems the sulfonate groups of PSS dominate cation exchange while the PEDOT backbone mainly sets electronic behavior. These differences imply that “CP” should not be treated as a single design variable: nitrogen-rich CPs act primarily through coordinative and acid-base interactions, whereas PEDOT-based systems act largely through their polyanionic dopant phase.

The oxidation (doping) state modulates electronic and ionic transport in opposite directions. Electronic conductivities of CPs span roughly 10^−6^–10^−3^ S cm^−1^ in weakly doped states and reach 10–10^2^ S cm^−1^ upon full doping (up to ≈10^3^ S cm^−1^ for optimized PEDOT:PSS), whereas stronger doping tightens ion pairing with dopant anions and can reduce ion mobility within the polymer phase. Dopant identity matters correspondingly: small mobile dopants (e.g., Cl^−^, ClO_4_^−^) support anion-exchange pathways but can leach and contribute to self-discharge, whereas large immobilized polyanions (e.g., PSS or sulfonate surfactants) fix the anionic charge and convert the CP phase into a cation-exchanging medium favorable for Zn^2+^ transport. For composite electrolytes and separator coatings, reported electronic percolation thresholds of CP fillers typically fall at a few weight percent (commonly ≈1–5 wt%, lower for high-aspect-ratio morphologies); CP loadings should therefore remain below this threshold, or the CP phase should be deliberately discontinuous above it, whenever device-scale electronic insulation is required.

Molecular weight, crystallinity, and conjugation length influence Zn^2+^ transport mainly indirectly, through film morphology: higher crystallinity and longer conjugation favor electronic transport but reduce free volume and water uptake, while amorphous, hydrated regions carry most of the ionic flux. In electrolyte and separator components, polaron and bipolaron states are therefore relevant not as charge carriers to be maximized, as in electrodes, but as the microscopic origin of two component-level phenomena: localized redox buffering, which can be beneficial, and electronic leakage along percolated pathways, which must be suppressed. Quantitative structure–transport relations for Zn^2+^ specifically remain sparse, and systematic measurements of Zn^2+^ transference numbers as a function of doping state and dopant identity are a research priority (Table in Section 4.3).

### 2.5. Design Strategies for CP-Based Electrolytes

**Figure 7:**
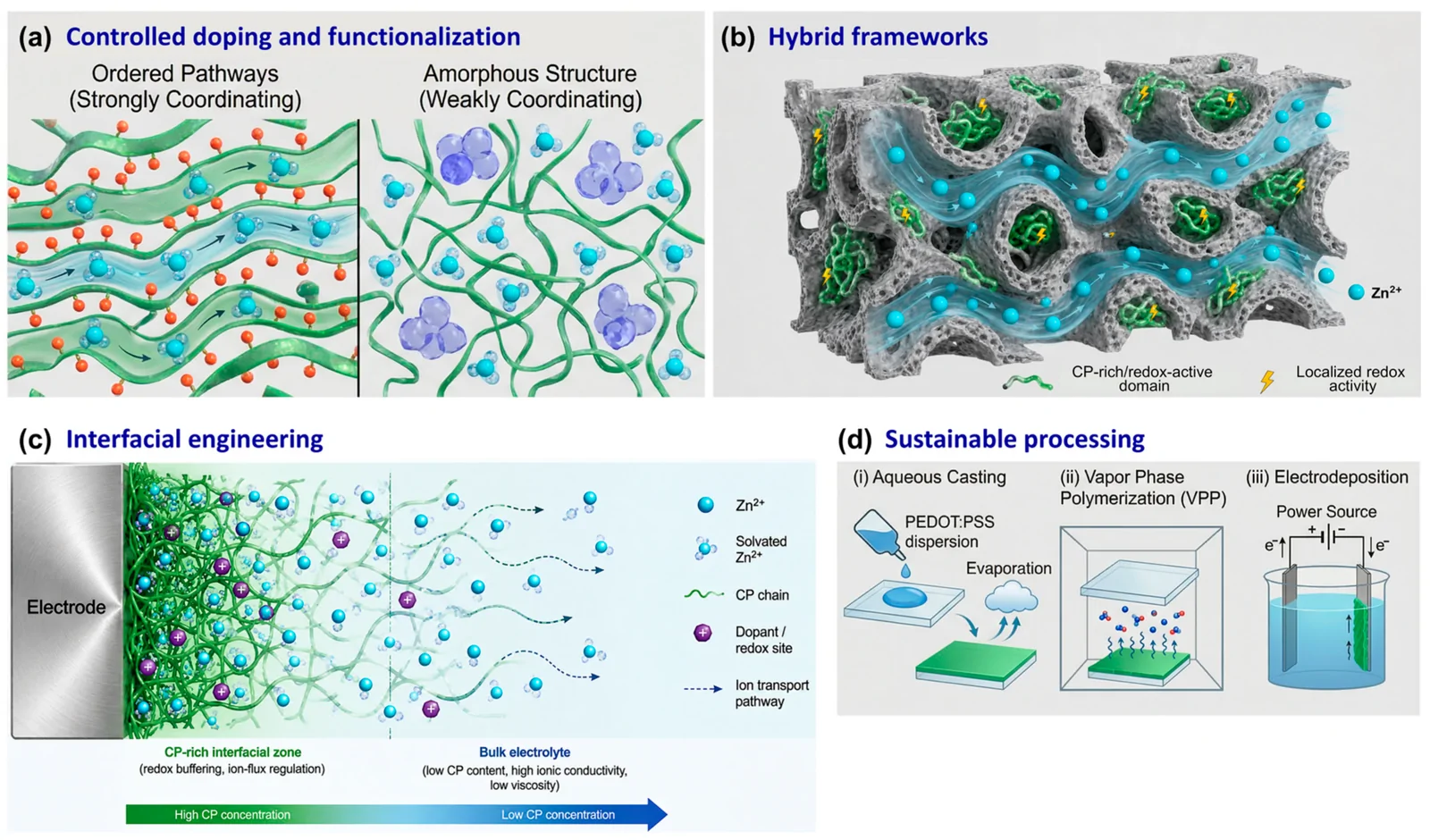
Design strategies for CP-containing electrolytes. (**a**) Controlled doping and functionalization. Dopant and side-chain chemistry regulate ion coordination, solvation, and segmental mobility in CP-containing electrolyte environments. Hydrophilic and strongly coordinating groups, such as –SO_3_^−^, can provide local Zn^2+^ coordination sites and guide hydrated Zn^2+^ transport. Bulky and weakly coordinating anions, such as bis(trifluoromethanesulfonyl)imide (TFSI^−^), can promote salt dissociation and free-volume-assisted ion mobility, although excessive weakening of specific coordination may reduce ion selectivity. (**b**) Hybrid electrolyte frameworks. Ion-permeable inorganic or porous frameworks, such as MOF or SiO_2_-based domains, can be combined with localized CP-rich domains to balance mechanical stability, ion transport, and redox buffering. The CP-rich domains should remain electronically limited or spatially discontinuous to suppress long-range electronic percolation, leakage current, and self-discharge. (**c**) Interfacial engineering. A gradient of CP concentration can be introduced across the electrolyte phase, with higher CP-derived redox activity near the electrode for local potential buffering and ion-flux regulation. The bulk electrolyte maintains lower CP content to preserve ion-dominated transport, low viscosity, and controlled electronic coupling. (**d**) Sustainable and scalable processing. Aqueous dispersion casting, vapor-phase polymerization, and electrodeposition provide representative routes for forming CP-containing electrolyte or interfacial architectures with controlled thickness, composition, and morphology. Selection of processing routes should consider solvent use, dopant retention, reproducibility, and compatibility with Zn-ion hybrid capacitor cell assembly.

Control of dopant chemistry and side-chain functionality in CPs such as PEDOT and PANI provides a molecular strategy for regulating ionic conductivity, redox potential, and compatibility with electrolytes. In aqueous Zn^2+^ electrolytes, hydrophilic and coordinating anions or pendant groups can influence water uptake and local coordination of hydrated Zn^2+^. Representative examples include sulfonate- and phosphonate-containing groups. Weakly coordinating or sterically bulky dopants can reduce ion binding strength and facilitate ion motion, whereas excessive weakening of coordination may reduce ion selectivity. For electrolytes containing CPs in Zn-ion hybrid capacitors, negatively charged side chains and weakly coordinating dopants can therefore be selected to form Zn^2+^-compatible transport environments. The oxidation state and redox potential of the CP backbone also depend on the electron-withdrawing character of the dopant and the local solvation environment, as illustrated in Figure 7a [87].

A second design strategy involves incorporation of CP domains into polymer-inorganic hybrid frameworks. In hybrid frameworks, the CP phase should provide localized redox-response sites and ion-interacting domains, rather than continuous pathways of electron transport. A rigid or porous scaffold, such as a metal–organic framework (MOF), silica network, or other inorganic matrix, can provide dimensional stability and Zn^2+^-permeable transport channels. For Zn^2+^ electrolyte systems, spatial division of function is useful because ion transport, local redox regulation, and resistance to swelling can be assigned to different structural domains. The design target is not long-range electronic conduction through the electrolyte. The target is controlled Zn^2+^ transport with confined redox activity and limited electronic leakage. The framework-level division of function is schematically represented in Figure 7b [2,90].

Interfacial engineering provides a third strategy by controlling the composition of the electrolyte near the electrode. This approach differs from the use of a separate continuous CP coating layer. Electrolytes containing CPs can be formulated with gradients in CP content, dopant concentration, redox activity, and hydrophilicity. For example, a CP-enriched interfacial region near the electrode may provide local redox buffering and regulation of ion flux. A less interactive bulk electrolyte region can preserve ionic conductivity and reduce viscosity. The graded design can confine redox activity to the electrode-adjacent region and reduce unnecessary electronic coupling across the electrolyte. The resulting electrolyte structure may reduce concentration polarization and limit parasitic interfacial reactions, as depicted in Figure 7c [90,91].

From a processing perspective, electrolytes containing CPs are compatible with several scalable and solvent-reduced fabrication routes. PEDOT:PSS can be processed from aqueous dispersions. Oxidative polymerization in water, vapor-phase polymerization, and electrodeposition provide additional routes for preparing CP-containing domains with controlled composition and interfacial conformity. These methods can reduce reliance on large volumes of harsh organic solvents. For Zn-ion hybrid capacitors, reproducible control over CP distribution, domain thickness, and interfacial contact is necessary. Processing should also prevent the formation of unintended continuous electronic pathways, especially when CP-rich regions are placed near separators or electrode–electrolyte interfaces. Representative processing routes are summarized in Figure 7d [92].

The design space of electrolytes containing CPs for Zn^2+^ energy-storage systems can be organized into four connected levels. The first level is molecular control of dopants and side chains. The second level is construction of polymer-inorganic hybrid frameworks. The third level is engineering of graded electrode–electrolyte interphases. The fourth level is solvent-reduced processing of architectures with defined CP distribution. The chemistry of doping, topology of the framework, structure of the interphase, and fabrication route jointly determine Zn^2+^ coordination, ion mobility, redox accessibility, swelling behavior, and dimensional stability [87]. For Zn-ion hybrid capacitors, electrolyte design should therefore optimize Zn^2+^ transport and local redox regulation while suppressing electronic leakage, self-discharge, and uncontrolled swelling of CP-containing domains.

### 2.6. Summary of Electrolyte-Level Functions

When incorporated into liquid, gel, or solid electrolyte systems, CPs can introduce functions beyond passive ion conduction. The first function is local ion-electron coupling. Conjugated polymer backbones can accommodate electronic or polaronic carriers, whereas ions migrate through polar, ionic, or solvating sites in CP-containing domains [87,93]. The ion motion maintains local electroneutrality during redox-state changes in CPs. For electrolyte applications, however, ion-electron coupling should not be interpreted as long-range electronic conduction across the cell. Excessive electronic connectivity can increase leakage current and self-discharge.

The second function is distributed redox buffering. CP-containing gels or electrolyte domains can participate in reversible charge compensation when redox-active sites remain chemically stable and spatially controlled [72]. Local redox buffering can moderate charge accumulation and reduce sharp potential gradients near electrode interfaces. The same redox activity may also become a failure factor if redox species diffuse freely or form shuttle pathways. Therefore, redox-active electrolyte designs require evaluation of redox reversibility, retention of polymeric redox species, leakage current, self-discharge behavior, and Coulombic efficiency.

The third function is chemical regulation of ion solvation and transport. Fixed anionic groups and polar functionalities in CP-containing electrolyte networks can modify the coordination environment of Zn^2+^. The functional groups may promote salt dissociation and guide Zn^2+^ migration through preferential pathways. In Zn-based gel and hydrogel electrolytes, sulfonate-containing or other polyanionic networks have been reported to reorganize Zn^2+^ solvation, accelerate ion transport and desolvation, and improve the reversibility of Zn deposition [90,91,94,95]. The electrolyte chemistry can therefore affect ion-transfer kinetics, rather than serving only as an ion-conducting medium.

CP-containing electrolyte networks can also contribute to mechanical stability. Polymer gel networks can preserve structural coherence during swelling, de-swelling, bending, and repeated charge–discharge operation [90,94,96,97]. When ion-regulating groups are combined with stable electrode–electrolyte contact, the electrolyte structure can reduce impedance growth, cracking, delamination, and deformation. The mechanical role should be evaluated together with swelling ratio, electrolyte uptake, modulus retention, and dimensional stability. Excessive swelling of CP-containing domains can impair ion selectivity and interfacial contact during long-term cycling.

A further electrolyte-level function is regulation of the interfacial environment. Ion occupancy, redox state, and local transport pathways can change together during operation. Controlled coupling among these variables may redistribute Zn^2+^ flux, moderate concentration polarization, and stabilize interfacial resistance. The effect is particularly relevant to Zn^2+^ systems because local solvation structure and uniformity of Zn deposition strongly influence cycling stability. Direct evidence should be obtained from Zn-ion hybrid capacitor cells when claims are made for Zn-ion hybrid capacitor performance.

Across these functions, electrolytes containing CPs should be described as ion-conducting media with localized redox and ion-regulating domains. The key design target is to regulate Zn^2+^ transport, local potential distribution, and interfacial stability while suppressing electronic leakage, redox shuttle behavior, uncontrolled swelling, and self-discharge. The following section extends electrolyte-level concepts to separator-related architectures. In separator environments, CP-containing domains must be spatially confined and electronically interrupted so that ion flux and deposition morphology can be regulated without forming continuous electron-transport pathways between electrodes [91,93].

Before CP functions are extended from electrolyte regulation to separator-related architectures, shared design constraints must be defined. CP domains can support ion coordination, redox buffering, and interfacial regulation, whereas insufficient confinement can cause electronic leakage, self-discharge, pore blockage, swelling, or chemical instability. Table 1 summarizes the validation criteria needed for balanced interpretation of CP-containing electrolytes and separator-related architectures in Zn-ion hybrid capacitors.

## 3. CPs in Separator Systems

In hybrid capacitors, the separator is the region where ion transport across the cell, local electric-field distribution, and mechanical confinement are coupled. The primary role of the separator remains electronic insulation together with ionic permeability. Any discussion of CPs in separator systems should therefore begin from a clear design constraint: CPs are not treated here as continuous electronically conducting separator films. Instead, CPs are considered as spatially confined interfacial layers, partial coatings, or electronically discontinuous domains incorporated into hybrid separator architectures.

**Figure 8:**
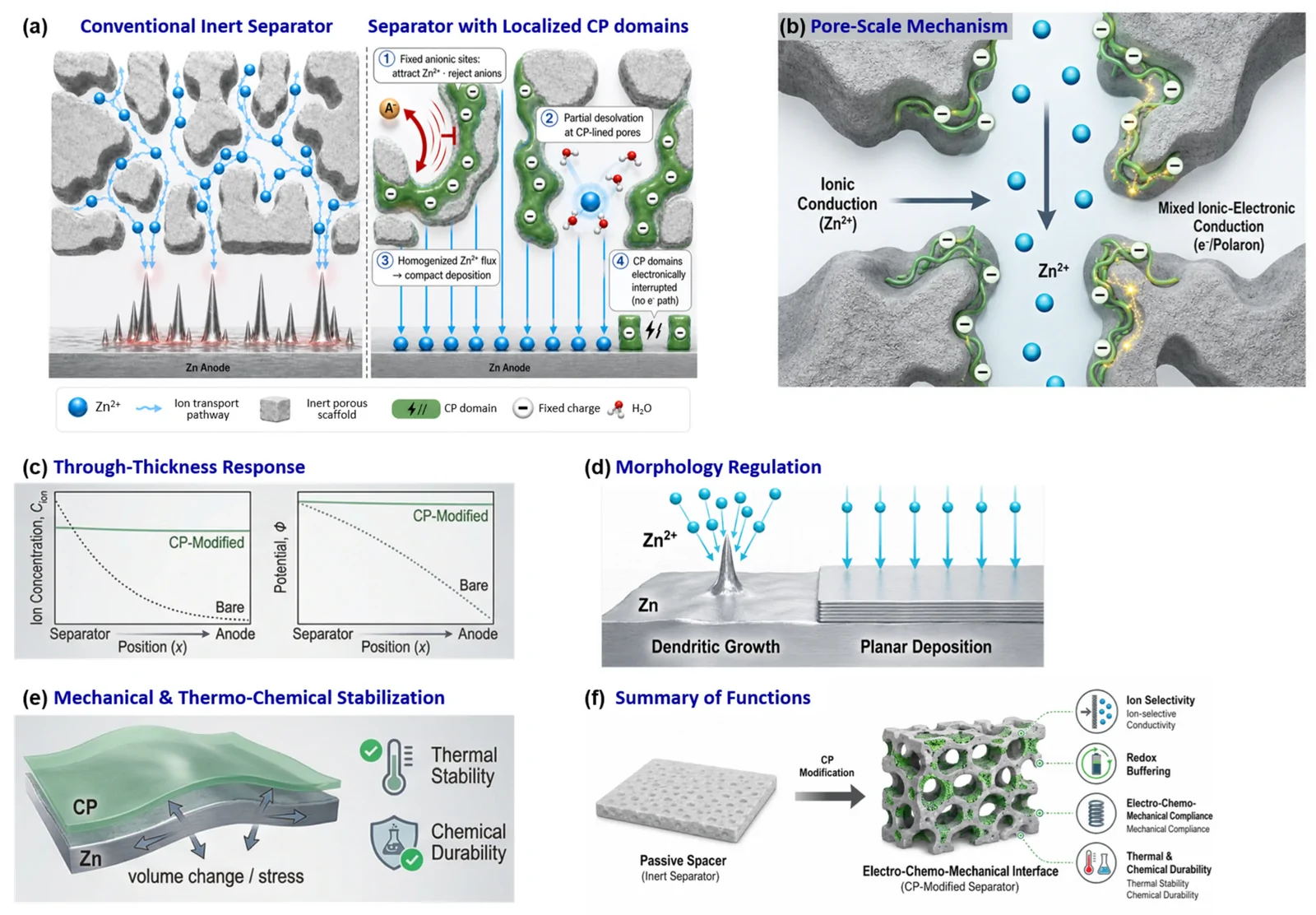
Separator-related architectures containing CP domains as multifunctional regulators in hybrid capacitors, with Zn^2+^-based systems shown as a representative example. (**a**) Comparison of a conventional inert separator and an insulating separator scaffold containing localized CP domains. Spatially confined and electronically interrupted CP domains regulate Zn^2+^ flux, local charge accumulation, and deposition uniformity without forming continuous electron-transport pathways, with numbered callouts indicating (1) fixed anionic sites that attract Zn^2+^ and reject anions, (2) partial desolvation at CP-lined pores, where coordinating CP sites displace part of the [Zn(H_2_O)_6_]^2+^ hydration shell, (3) homogenized Zn^2+^ flux leading to compact deposition, and (4) electronic discontinuity of the CP domains. (**b**) Pore-scale mechanism showing fixed charges, dopant-controlled coordination environments, pore accessibility, and local redox buffering in CP-containing domains. (**c**) Through-thickness response showing reduced concentration *C*_ion_(*x*) and potential *Φ*(*x*) gradients relative to an inert separator. (**d**) Morphology regulation at the Zn interface, where weakened field focusing and more uniform Zn^2+^ arrival suppress dendritic growth and promote planar Zn deposition. (**e**) Mechanical and thermo-chemical stabilization through stress distribution, pore retention, puncture resistance, and improved tolerance to thermal or chemical stress. (**f**) Summary of separator-level functions, including ion-selective transport, local redox buffering, electro-chemo-mechanical compliance, and durability, while preserving electronic insulation.

Compared with inert porous membranes, separator architectures containing CPs can introduce fixed-charge sites, polar functional groups, and localized redox-response domains at pore surfaces or near the electrode-facing side of the separator. The resulting chemical heterogeneity can modify the local coordination environment of Zn^2+^, the surface polarity of pore walls, and the distribution of electrostatic potential across the separator. In Zn^2+^ hybrid systems, regulation of the local separator environment is particularly important because nonuniform flux of Zn^2+^ through inert membranes can intensify current concentration, interfacial crowding, and dendritic or filamentary Zn growth (Figure 8a). As highlighted by callouts (1)–(4) in Figure 8a, localized CP domains counteract these failure modes through four coupled actions: (1) fixed anionic sites that attract Zn^2+^ and reject anions; (2) partial desolvation at CP-lined pores; (3) homogenization of the Zn^2+^ flux toward compact deposition; and (4) electronic discontinuity of the CP domains, which prevents through-plane electron transport. In step (2), the desolvating species is the hydrated [Zn(H_2_O)_6_]^2+^ cation: sulfonate and amine sites on the pore walls substitute for one or two coordinated water molecules, so that part of the desolvation penalty—normally paid entirely at the Zn surface, where the released interfacial water can feed hydrogen evolution—is pre-paid inside the separator. This lowers the remaining interfacial activation barrier (cf. Figure 4c) and moves water release away from the deposition front.

The behavior of separator architectures containing CPs depends on the chemistry, spatial distribution, and continuity of the CP phase. Fixed-charge density, polarity, dopant distribution, and accessible redox state can influence the coordination microenvironment encountered by migrating Zn^2+^, the degree of electric-field screening near pore walls, and the balance between ion guidance and pore accessibility (Figure 8b). The most useful separator designs therefore regulate ion flux without obstructing ionic transport and without creating continuous pathways of electron transport across the separator.

Under high-rate or high-areal-capacity conditions, localized CP domains may also buffer transient changes in ion concentration and interfacial charge across the separator thickness. Local charge compensation and confined redox equilibration may reduce field amplification near protrusions and moderate concentration gradients relative to inert membranes. The resulting interfacial conditions may support more uniform Zn deposition and suppress localized depletion or accumulation of ions (Figure 8c,d). However, the same CP domains may increase leakage current or self-discharge when coating thickness, domain connectivity, or electronic percolation are not properly controlled [19,98,99].

Separator architectures containing CPs may also contribute to physicochemical stabilization. CP-containing hybrid structures and compliant interfacial layers may distribute stress generated during Zn plating and stripping, improve resistance to pore deformation or puncture, and support thermal or chemical durability. The separator must nevertheless remain electronically insulating at the device level. Evaluation of CP-containing separator architectures should therefore include ionic resistance, leakage current, self-discharge behavior, dimensional stability, puncture resistance, and cycling durability, rather than ion-flux regulation alone (Figure 8e,f).

Electronic insulation itself requires explicit quantification. Bulk electronic conductivity of a CP-containing membrane is necessary but not sufficient: device-relevant screening should combine (i) DC polarization of electrode/separator/electrode stacks to extract the electronic leakage current at operating voltage, (ii) voltage-hold (float-current) and open-circuit self-discharge tests in full cells, and (iii) post-cycling verification that no percolated electronic pathway has formed. These cell-level measurements, rather than electronic conductivity alone, define whether localized CP domains remain acceptably interrupted in operation, and they underlie the validation criteria of Table 1.

Within these constraints, CPs in separator systems should be discussed as separator-supported and architecture-controlled interfacial domains. The value of CP incorporation lies in regulation of Zn^2+^ flux, moderation of local electric-field heterogeneity, and stabilization of separator-electrode interfaces, provided that continuous electronic pathways are avoided.

### 3.1. Separator-Supported Interfacial Layers for Ion Selectivity and Stability

Separator-supported CP-containing interfacial layers have been introduced to polyolefin, cellulose-based, and glass-fiber separators to regulate ion selectivity and interfacial stability [100,101,102]. These architectures preserve the electronically insulating separator scaffold while introducing localized CP-containing domains at selected separator interfaces. CP-containing domains can be positioned as thin interfacial regions, pore-wall domains, or partial functional layers on insulating separator supports. The polarity of the CP backbone and the chemistry of dopant anions, including sulfonate groups, can tune surface potential and local coordination environments near separator pores. The resulting fixed-charge sites and polar groups can bias the distribution and flux of solvated Zn^2+^ near the metal interface.

**Figure 9:**
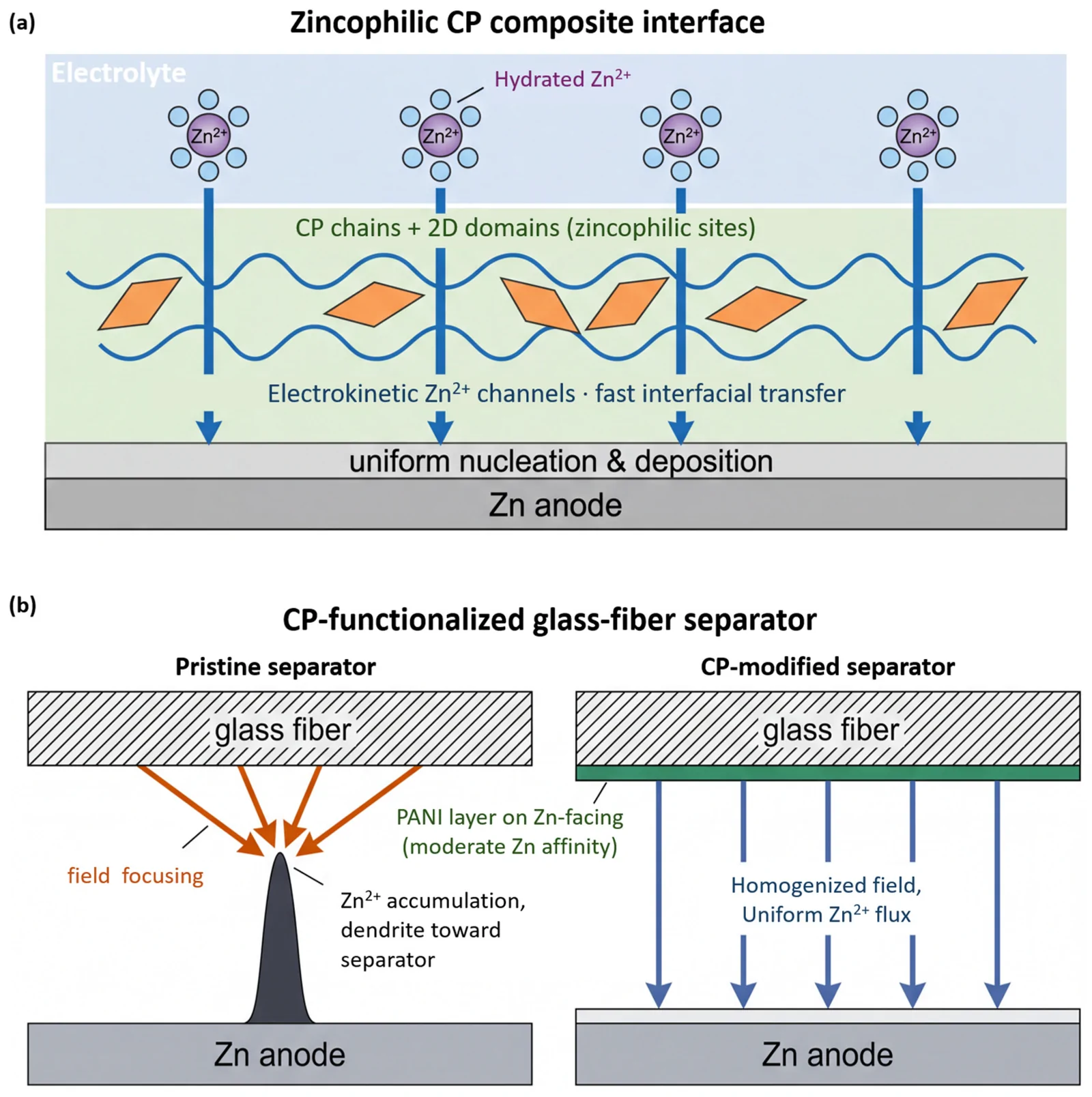
Schematic illustration of CP-containing interfacial layers for Zn^2+^ flux regulation. (**a**) Zincophilic CP composite interface on the Zn anode: a CP layer combined with two-dimensional (2D) domains provides electrokinetic Zn^2+^ transport channels, accelerates interfacial Zn^2+^ transfer, and guides uniform nucleation on the Zn surface. (**b**) CP-functionalized glass-fiber separator: a pristine separator promotes nonuniform electric-field distribution and dendrite growth toward the separator, whereas a PANI-containing interfacial layer on the Zn-facing side provides moderate Zn affinity, weakens Zn-separator binding, homogenizes the local field, and guides smoother Zn deposition. The schematics were redrawn based on the design concepts reported by Wang et al. [104] and Wu et al. [106].

Mechanistic support for fixed-anion control of Zn deposition has also been obtained from ionomer-type separator studies. Sulfonate-rich Nafion architectures and Zn^2+^-integrated Nafion membranes regulated Zn^2+^ transport across separator structures and promoted smoother Zn deposition [100,103]. A closely related anode-side artificial interface combined PEDOT:PSS with monolayer MoS_2_ [104]. In that design, zincophilic PEDOT was reported to accelerate Zn^2+^ transfer kinetics and improve deposition uniformity. The proposed mechanism involved electrokinetic channel-type regulation of Zn^2+^ transport (Figure 9a). Although this interface was constructed directly on the Zn anode rather than on a separator, it demonstrates how a localized CP-containing layer can regulate interfacial ion flux, a principle transferable to separator-supported designs.

As an electrostatic analog, cationic polymer functional layers in Li-S cells have been shown to confine anionic polysulfides while maintaining cation conduction [105]. The Li-S example supports the broader principle that fixed charges at separator interfaces can gate ion transport and stabilize interfacial chemistry. The relevance of Li-S separator studies to Zn-ion hybrid capacitors is limited to electrostatic design principles because Zn^2+^ transport, hydration, desolvation, and plating chemistry differ from monovalent-cation and polysulfide-containing systems.

Direct separator-level evidence has been reported in Zn systems. Wu et al. [106] modified one side of a commercial glass-fiber (GF) separator by in situ PANI formation to prepare a GF/PANI separator, with the PANI-modified face oriented toward the Zn anode. The unmodified glass-fiber separator produced a nonuniform electric-field distribution at the Zn surface. Strong binding between glass fiber and Zn also biased Zn^2+^ accumulation toward the separator, increasing the risk of dendrite penetration. In contrast, the PANI-containing interfacial layer provided polar functional groups with moderate Zn affinity. The modified interface guided Zn^2+^ deposition along the edges of nascent Zn nuclei and produced smoother plating. The optimized GF/PANI-600 separator was therefore described as balancing Zn affinity with a more uniform field distribution, leading to more homogeneous Zn deposition (Figure 9b).

From a mechanical and morphological viewpoint, ultrathin CP-containing layers on insulating separators can be compared with thin ion-exchange or gel polymer coatings [107]. When the layer thickness remains controlled, these interfacial domains can reinforce pore walls, improve electrolyte affinity, and stabilize pore geometry without blocking ion transport. Electronic insulation at the device level must still be verified because excessive CP loading or domain connectivity can increase leakage current and self-discharge. Relevant evaluations should therefore include ionic resistance, leakage current, self-discharge rate, electrolyte uptake, pore accessibility, and cycling stability.

Processing studies further show that conformal CP-containing layers can be prepared on porous separator supports. Vapor-phase polymerization has produced lightweight PEDOT layers on porous polyolefin separators while preserving pore connectivity in lithium metal cells [108,109]. This processing route provides a useful precedent for preparing thin CP-containing domains on separator substrates. Its relevance to aqueous Zn^2+^ hybrid capacitors should be framed as a processing analogy until Zn^2+^-based separator cells confirm ion selectivity, charge retention, and dendrite suppression. For Zn-based systems, reproducible control over CP loading, layer thickness, domain continuity, and electronic leakage is essential for separator-oriented design.

### 3.2. Self-Supported and Hybrid Membranes Containing CP Domains

Self-supported and hybrid membranes containing CP domains extend separator design beyond surface-modified porous substrates. However, a separator must remain electronically insulating at the device scale. Therefore, CP-containing membranes should not be interpreted as pure free-standing conducting polymer films. Their practical use requires control over long-range electronic percolation to avoid leakage current, self-discharge, and internal shorting. Relevant designs include insulating polymer frameworks containing dispersed CP domains, fixed-anion membranes with ion-selective sites, and hybrid membranes in which CP-rich regions are spatially confined.

The fixed-anion-site concept provides a mechanistic basis for Zn^2+^ regulation in membrane-type separators. Sulfonate-rich ionomer membranes, including Nafion-type architectures, regulated Zn^2+^ transport and homogenized ion flux across separator structures [103,110]. The more uniform flux of Zn^2+^ promoted smoother Zn deposition. The same design principle can guide the use of sulfonate-bearing CP motifs, including PEDOT:PSS-like anionic domains, for separator-level ion regulation. The analogy should remain limited to ion-selective transport, unless direct measurements confirm electronic insulation, leakage-current suppression, and cycling stability in Zn-ion hybrid capacitor cells.

Hybrid CP-containing membranes provide a more practical route than pure CP films. In these architectures, CP or polyelectrolyte domains are embedded in mechanically stable insulating frameworks, including cellulose, chitosan, or PVDF-class matrices [111,112,113,114]. The scaffold preserves dimensional stability, pore accessibility, and ion-permeable pathways during cycling. CP or polyelectrolyte domains introduce fixed charges and polar coordination sites. Inorganic nanofillers, including SiO_2_, Al_2_O_3_, or MXene, can further tune the distribution of Zn^2+^ and the mechanical resistance of the membrane. The intended role of the CP phase is ion-flux regulation and local interfacial control, not continuous electronic conduction through the separator.

**Figure 10:**
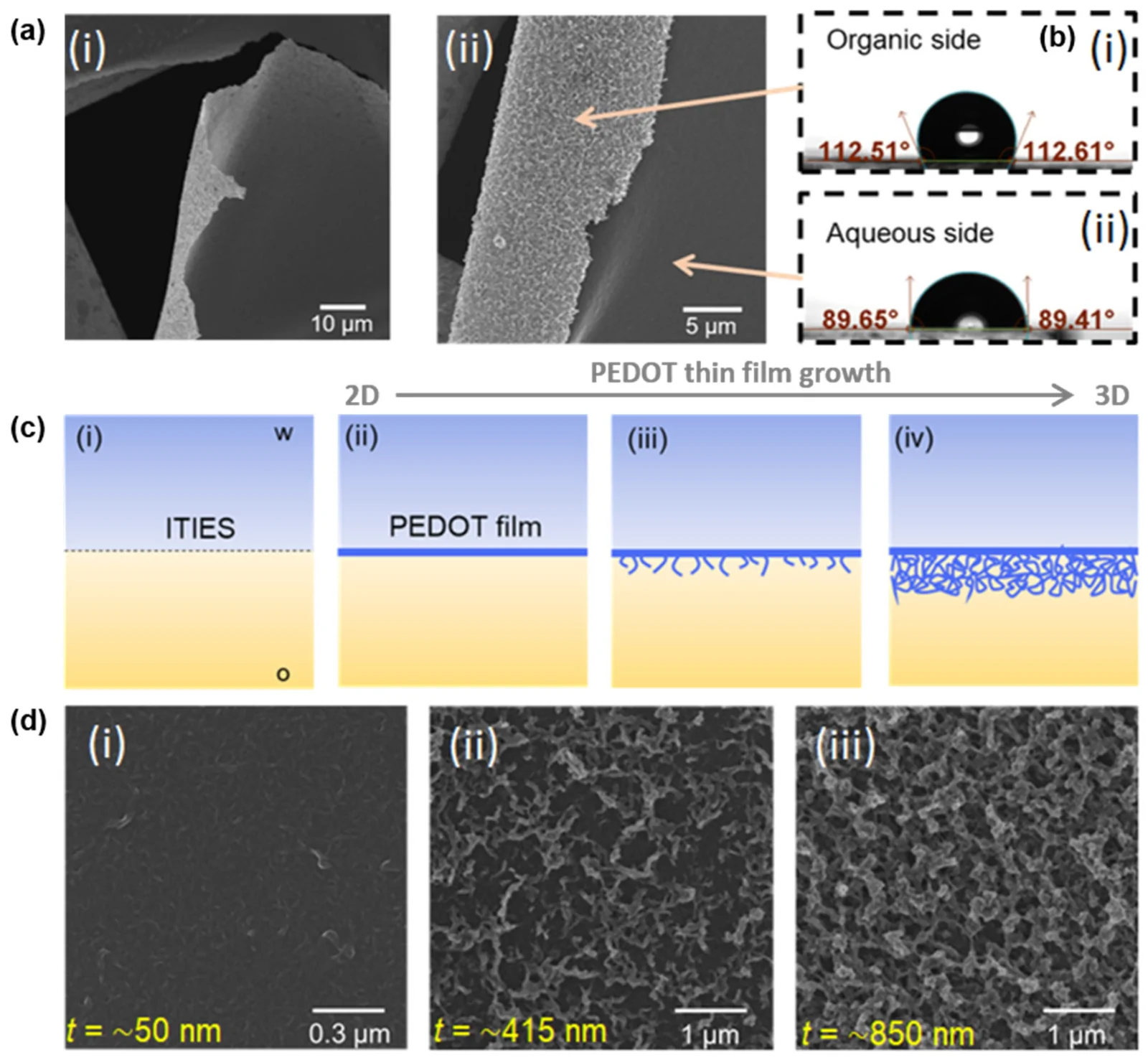
Representative free-standing CP membrane architectures for Zn^2+^ separator design. Free-standing PEDOT thin films with tunable morphology and growth mode: (**a**) Scanning electron microscopy (SEM) images of a free-standing 2D PEDOT film electrosynthesized at the interface between two immiscible electrolyte solutions (ITIES), showing the distinct morphologies of the “smooth” aqueous-facing side and “rough” organic-facing side. (**b**) Sessile drop contact angle measurements recorded on each side of the PEDOT thin film, highlighting the influence of the morphology on the measured hydrophobicity. (**c**) Schematic of the different stages of PEDOT thin film growth from 2D to 3D as a function of continued double-potential step chronoamperometry (DPSCA) cycling. (**d**) SEM images showing the organic-facing side of the PEDOT thin film, demonstrating the controllable growth on the organic-facing side of the thin film from 2D to 3D as a function of DPSCA cycling. The thickness (*t*) of each thin film was determined by atomic force microscopy (AFM). Reproduced from [115] under the terms of the Creative Commons CC BY 4.0 license. Copyright 2022, The Authors, published by American Chemical Society.

Control over morphology is essential for self-supported membranes containing CP domains. Free-standing CP films prepared for electrochemical platforms have shown that thickness, surface topology, and growth mode can be tuned by electrochemical synthesis [115]. Reported structures ranged from compact 2D films to three-dimensional (3D) fibrous networks (Figure 10a–d). For separator deployment, however, morphological control must be paired with insulation-aware design. The CP phase should not form an electronically continuous pathway between the electrodes. Measurements of leakage current, self-discharge rate, ionic resistance, and puncture resistance are therefore necessary.

A related strategy involves hybrid networks that combine conjugated polymer domains with mechanically supportive frameworks and secondary CP phases [116]. The resulting structures can provide flexibility, ion permeability, and resistance to mechanical deformation. Their relevance to separator systems depends on whether post-treatment or structural organization interrupts electronic percolation while preserving ion-accessible pores. Therefore, hybrid CP-containing membranes should be evaluated through both transport and safety metrics. Key parameters include Zn^2+^ transference number, ionic conductivity, dimensional stability, pore accessibility, leakage current, self-discharge behavior, and cycling retention.

Self-supported separator membranes containing CP domains are best discussed as insulating or weakly electronically connected hybrid membranes with localized ion-regulating motifs. Pure conducting polymer films are unlikely to satisfy separator requirements without additional structural control. The most defensible design direction is to combine fixed-charge CP motifs, insulating support frameworks, and controlled pore structures to regulate Zn^2+^ transport while preventing long-range electron transport across the separator.

### 3.3. Dendrite Suppression and Electric-Field Homogenization

**Figure 11:**
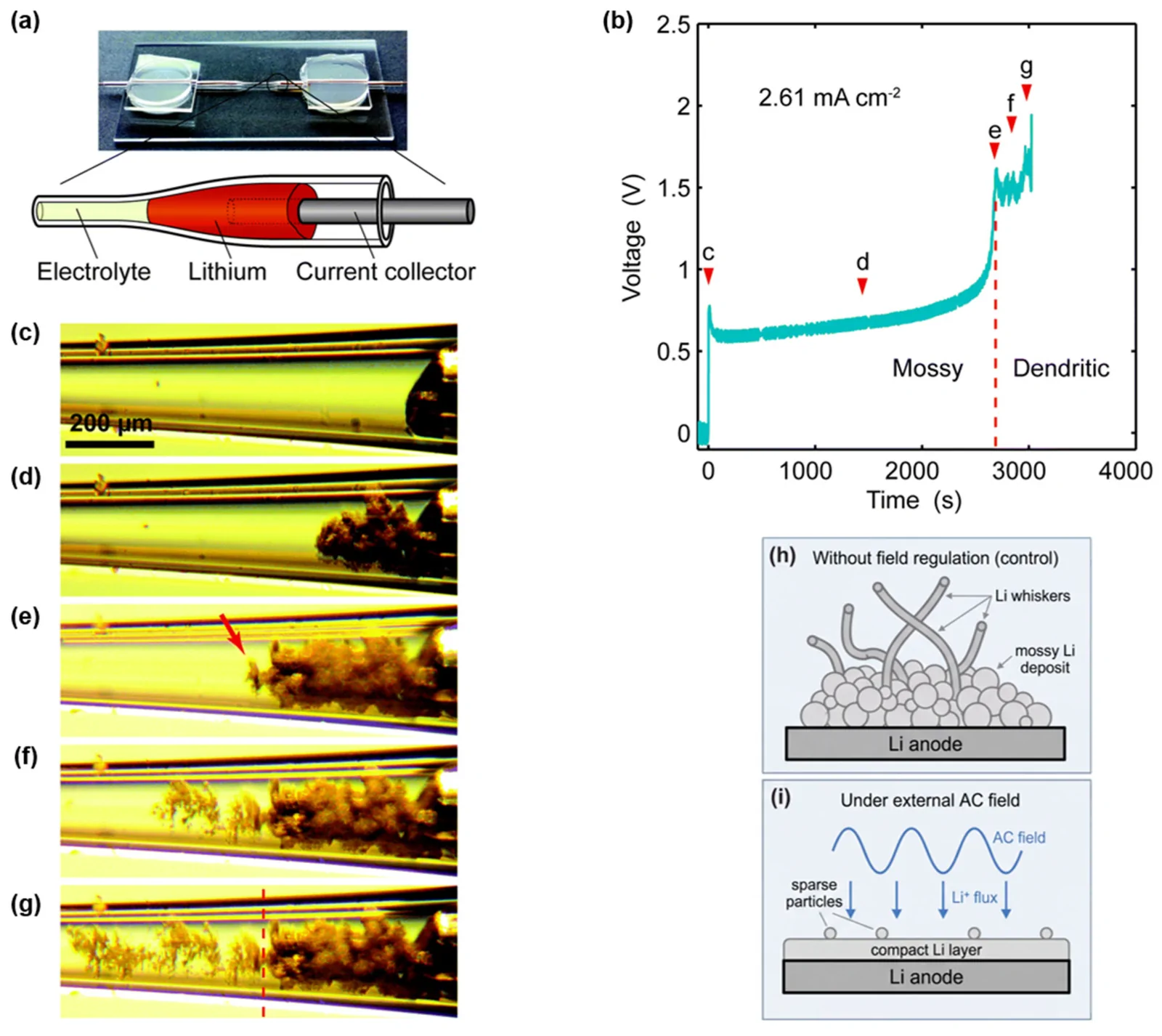
Electrostatic origin of dendrite growth during metal electrodeposition and its suppression by enhanced interfacial ion transport. (**a**–**g**) In situ observations of lithium electrodeposition in a glass capillary filled with an electrolyte solution consisting of 1 M LiPF_6_ in ethylene carbonate/dimethyl carbonate (EC/DMC): (**a**) Photo of the capillary cell, whose middle part was pulled thinner for easier optical observation. (**b**) Voltage responses of the capillary cell at a deposition current density of 2.61 mA cm^−2^. (**c**–**g**) In situ snapshots of the growth of lithium during the electrodeposition. The red arrow in (**e**) points to the emergence of dendritic lithium, and the red dashed line in (**g**) marks the clear morphological difference between the lithium deposits formed before and after Sand’s time. Reproduced from [117] under the terms of the Creative Commons CC BY-NC 3.0 license. Copyright 2016, The Authors, published by the Royal Society of Chemistry. (**h**,**i**) Schematic representation of the cycled-anode morphologies reported for this system: (**h**) without field regulation, the cycled lithium surface develops a rough, mossy deposit carrying an entangled network of lithium whiskers, whereas (**i**) under the external alternating current field the surface remains flat, forming a compact deposit with only sparse rounded particles. Panels (**h**,**i**) are schematic illustrations, not micrographs, drawn to summarize the observations of Chen et al. [118].

Beyond providing ion selectivity and redox activity, CP domains integrated into the separator can play a critical role in modulating the local electric-field distribution at the anode surface. Dendritic metal growth is fundamentally initiated by electric-field amplification at nanoscale surface protrusions. For instance, Bai et al. [117] demonstrated in a lithium-metal model system that space-charge accumulation near such asperities promotes preferential ion migration, leading to unstable filamentary deposition. The electrostatic origin of this instability—the coupling of local charge accumulation with field amplification—is illustrated in Figure 11a–g. Although the analysis by Bai et al. was developed for lithium metal, their findings serve as a crucial mechanistic analog, rather than as direct evidence, for Zn-metal anodes. The electrostatic boundary conditions are broadly relevant to any metal–electrolyte interface where field-driven ion migration contributes to deposition. Zn-based systems, however, introduce significant additional complexities, including Zn^2+^ hydration/desolvation dynamics, parasitic corrosion, hydrogen evolution, and distinct plating chemistry. Consequently, translating the Li-based field-amplification model to Zn-ion systems requires rigorous, Zn-specific experimental and theoretical validation.

Electric-field amplification at surface protrusions can intensify local current density and promote nonuniform metal deposition. The resulting surface roughening, in turn, increases the probability of parasitic reactions and dendrite penetration. Moderation of the interfacial field gradient is therefore linked to morphology stabilization. Relevant outcomes include reduced protrusion growth, lower current focusing, and improved uniformity of metal deposition. The electrostatic contribution should be considered together with electrolyte chemistry, ion solvation, and mechanics of the electrode–separator interface.

Separator engineering offers a practical route to regulate interfacial electric-field distribution. Complementary evidence for this design target comes from external-field control: Chen et al. [118]. enhanced lithium-ion diffusion in a lithium metal model system by applying external alternating and direct current fields, without modifying the cell materials or structure. Mitigating the diffusion limitation near the anode suppressed filamentary growth and promoted compact metal deposition (Figure 11h,i). Although realized with external fields rather than separator modification, the result identifies the same design target for separator architectures: homogenization of interfacial ion flux and local field distribution. For Zn-based systems, the study provides a useful design analog. Direct claims for Zn-ion hybrid capacitors should be supported by Zn plating-stripping data and full-cell cycling tests.

In the electrostatic-control framework, CP-containing interfacial domains on the Zn-facing side of separators may help redistribute local potential gradients. The CP domains should be spatially confined and electronically interrupted. Long-range electronic conduction across the separator thickness must be avoided to prevent leakage current, self-discharge, or internal shorting. Localized CP domains can provide fixed-charge sites, polar coordination environments, and confined redox response. These features may moderate field concentration near nascent Zn protrusions and reduce tip-accelerated growth.

Attenuation of local field amplification can decrease current focusing during Zn plating. A more uniform electrostatic driving force can favor compact Zn growth rather than mossy or needle-like morphologies. CP-containing separator interfaces may also reduce electro-chemo-mechanical feedback caused by uneven plating and stripping. The contribution of CP domains should be evaluated through field distribution analysis, Zn morphology, leakage current, self-discharge behavior, and long-term cycling stability. Comprehensive reviews on dendrite suppression provide broader context for the relationship among ion flux, electric-field distribution, and mechanical constraints [119].

Dendrite suppression by CP-containing separator architectures should be described as an electro-chemo-mechanical process. Electric-field homogenization, ion-flux regulation, morphology-controlled Zn deposition, and strain accommodation can contribute together to interfacial stabilization. The role of CPs is not to create an electronically conducting separator. The defensible design principle is to place CP-containing domains as localized ion-regulating and charge-buffering regions while preserving electronic insulation at the device scale. Mechanistic insights from Li metal systems can guide separator design, whereas validation in Zn-ion hybrid capacitors requires Zn-specific transport, deposition, self-discharge, and cycling data [117,118,119].

### 3.4. Thermal, Chemical, and Electrochemical Durability

**Figure 12:**
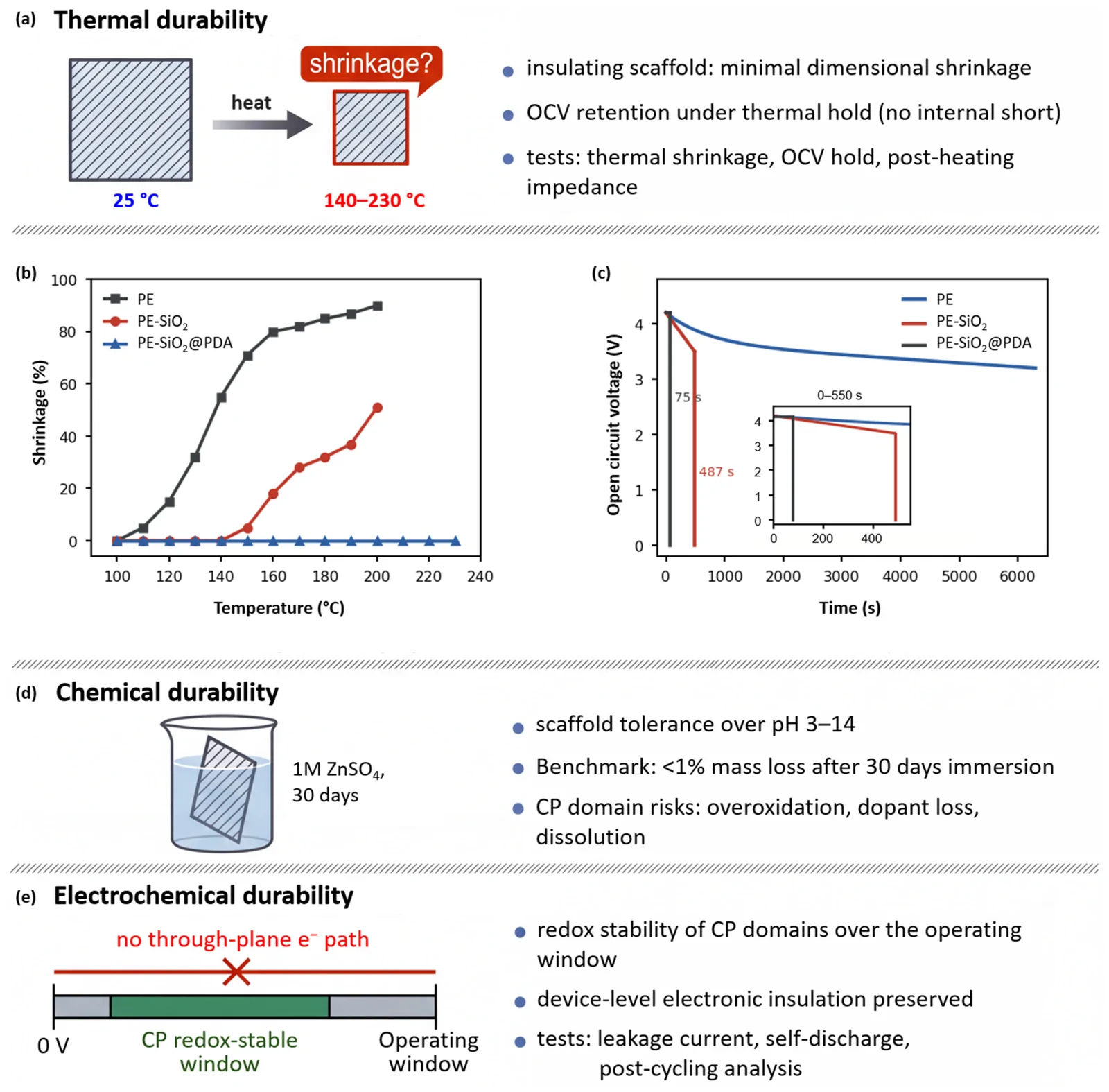
Durability benchmarks for separator architectures containing CP domains. (**a**) Thermal durability: the insulating scaffold should resist dimensional shrinkage at elevated temperatures, and safety-relevant evaluation includes thermal shrinkage tests, OCV hold measurements under heating, and post-heating impedance analysis (cf. Dai et al. [120] and Kang et al. [121]). (**b**) Thermal shrinkage of pristine PE, PE-SiO_2_, and PE-SiO_2_@PDA separators as a function of temperature: the PDA-SiO_2_ composite modification suppresses shrinkage up to 230 °C. (**c**) OCV of pouch cells assembled with the three separators during a 170 °C hold: cells with PE and PE-SiO_2_ separators show an abrupt voltage drop upon separator failure, whereas the PE-SiO_2_@PDA cell maintains a stable OCV (inset: enlarged early-time profiles). Panels (**b**,**c**) show the thermal-shrinkage and OCV data of [120], replotted for clarity and adapted under the terms of the Creative Commons CC BY 3.0 license. Copyright 2016, The Authors, published by the Royal Society of Chemistry. (**d**) Chemical durability: the scaffold and the CP-containing region should tolerate a wide pH range (pH 3–14) and prolonged exposure to aqueous Zn^2+^ electrolytes, with a practical benchmark of less than 1% mass loss after 30 days in 1 M ZnSO_4_, while avoiding overoxidation, dopant loss, and dissolution of CP domains (cf. Nian et al. [122]). (**e**) Electrochemical durability: CP-containing interfacial domains should remain redox-stable over the operating potential window while the separator preserves device-level electronic insulation, as verified by leakage current, self-discharge, and post-cycling analyses (cf. Su et al. [123] and Liu et al. [124]).

Thermal durability of separators remains a primary safety requirement because shrinkage of polyolefin membranes at elevated temperatures can promote direct electrode contact and internal short circuits (Figure 12a). Dai et al. [120] quantified thermal shrinkage of pristine polyethylene (PE), silicon dioxide-coated PE (PE-SiO_2_), and polydopamine (PDA)-modified SiO_2_-coated PE (PE-SiO_2_@PDA) separators. PE-SiO_2_@PDA showed negligible shrinkage up to 230 °C, whereas pristine PE exhibited pronounced shrinkage above 100 °C (Figure 12b). An open-circuit voltage (OCV) hold test at 170 °C further supported the safety relevance of dimensional stability. Rapid voltage collapse occurred for PE and PE-SiO_2_, whereas PE-SiO_2_@PDA maintained OCV during thermal exposure (Figure 12c). Although Dai et al. focused on Li-based cells, the result provides a separator-level safety benchmark for membrane shrinkage and short-circuit onset. The relevance to Zn-based hybrid capacitors should be treated as a materials-level analogy, not as direct Zn-cell evidence. Zn-based systems may use similar polyolefin backbones, although aqueous electrolytes introduce further requirements related to water uptake, corrosion, and interfacial chemistry. Separator architectures containing CP domains should therefore be evaluated for thermal shrinkage, OCV retention under thermal stress, and preservation of pore structure. Kang et al. [121] compared PE, 2-(dimethylamino)ethanethiol (DMAET)-modified PE (DMAET-PE), and bioinspired silica-coated PE (Silica-PE). Silica-PE showed reduced thermal shrinkage after exposure to 140 °C for 1 h relative to PE and DMAET-PE. OCV monitoring at 140 °C also confirmed separator-level safety. PE and DMAET-PE exhibited abrupt voltage collapse, whereas Silica-PE maintained stable OCV during the measurement period. SEM images indicated preservation of porous morphology after surface modification. Static water contact angles decreased from PE to DMAET-PE and further to superhydrophilic Silica-PE, linking surface chemistry with electrolyte wetting and separator performance. The above results of Dai et al. and Kang et al. define useful thermal shrinkage and OCV stability targets for separator architectures containing CP domains. For Zn-based systems, CP-containing interfacial regions should not compromise the dimensional stability of the insulating separator scaffold. The CP phase should also avoid excessive swelling or electronic percolation under thermal stress. Relevant tests include shrinkage measurements, thermal OCV holds, electrolyte uptake, ionic resistance after heating, leakage current, and self-discharge behavior.

Chemical durability imposes an additional constraint in aqueous Zn-based systems. Separator backbones must tolerate broad pH ranges and long exposure to hydrated Zn salts. Nian et al. [122] summarized that separator materials should tolerate pH 3–14 and reported a practical benchmark of less than 1% mass loss after 30 days in 1 M ZnSO_4_. Cellulose-based filter paper was described as vulnerable to degradation in strongly alkaline electrolytes such as KOH. Filter paper and glass-fiber separators illustrate the contrast between chemically vulnerable cellulose backbones and more durable inorganic fibrous matrices under corrosive electrolyte conditions. For separator architectures containing CP domains, chemical durability must be considered for both the host scaffold and the CP-containing region. The scaffold should resist hydrolysis, oxidation, and loss of mechanical integrity in the relevant electrolyte. The CP-containing domain should resist overoxidation, dopant loss, backbone cleavage, and dissolution of polymer fragments. Soluble degradation products may alter ion transport, increase self-discharge, or contaminate electrode surfaces. Therefore, chemical stability should be evaluated using mass retention, morphology retention, electrolyte contamination, ionic resistance, and cycling performance after electrolyte aging (Figure 12d).

Electrochemical durability requires that CP-containing interfacial domains remain stable over the relevant potential range while the separator maintains electronic insulation at the device level. Su et al. [123] reported operando evidence that a PEDOT coating inhibited surface feature formation and improved cycling stability of LiCoO_2_ over 3.0–4.5 V. Liu et al. [124] reported complementary evidence for a Ni-rich layered oxide platform cycled between 2.8 and 4.6 V. The study compared coated and uncoated single-crystal LiNi_0.83_Mn_0.10_Co_0.07_O_2_ after 200 cycles using ptychographic X-ray computed tomography, SEM, and transmission electron microscopy (TEM). The PEDOT-coated material exhibited reduced intragranular microcracking and mitigated surface reconstruction. However, since the studies by Su et al. and Liu et al. evaluated cathode coatings in Li-based cells, rather than separator-facing CP domains, their relevance to Zn-based separator architectures should be limited to oxidative stability and interfacial protection of selected CP chemistries. High-voltage Li platforms can provide conservative screening references for PEDOT and polythiophene derivatives. Direct application to Zn-ion hybrid capacitor separators requires separate validation of leakage current, self-discharge, electrolyte compatibility, and long-term cycling stability (Figure 12e).

Taken together, thermal, chemical, and electrochemical durability define mandatory screening criteria for separator architectures containing CP domains. The insulating separator scaffold must retain dimensional stability, pore accessibility, and chemical resistance. CP-containing regions must provide ion-regulating or interfacial functions without creating continuous electronic pathways. For Zn-based hybrid capacitors, credible evaluation should combine thermal shrinkage tests, OCV hold measurements, chemical aging, electrochemical stability tests, leakage-current analysis, self-discharge measurements, and long-term cycling data.

### 3.5. Design Principles for CP-Based Separators

**Figure 13:**
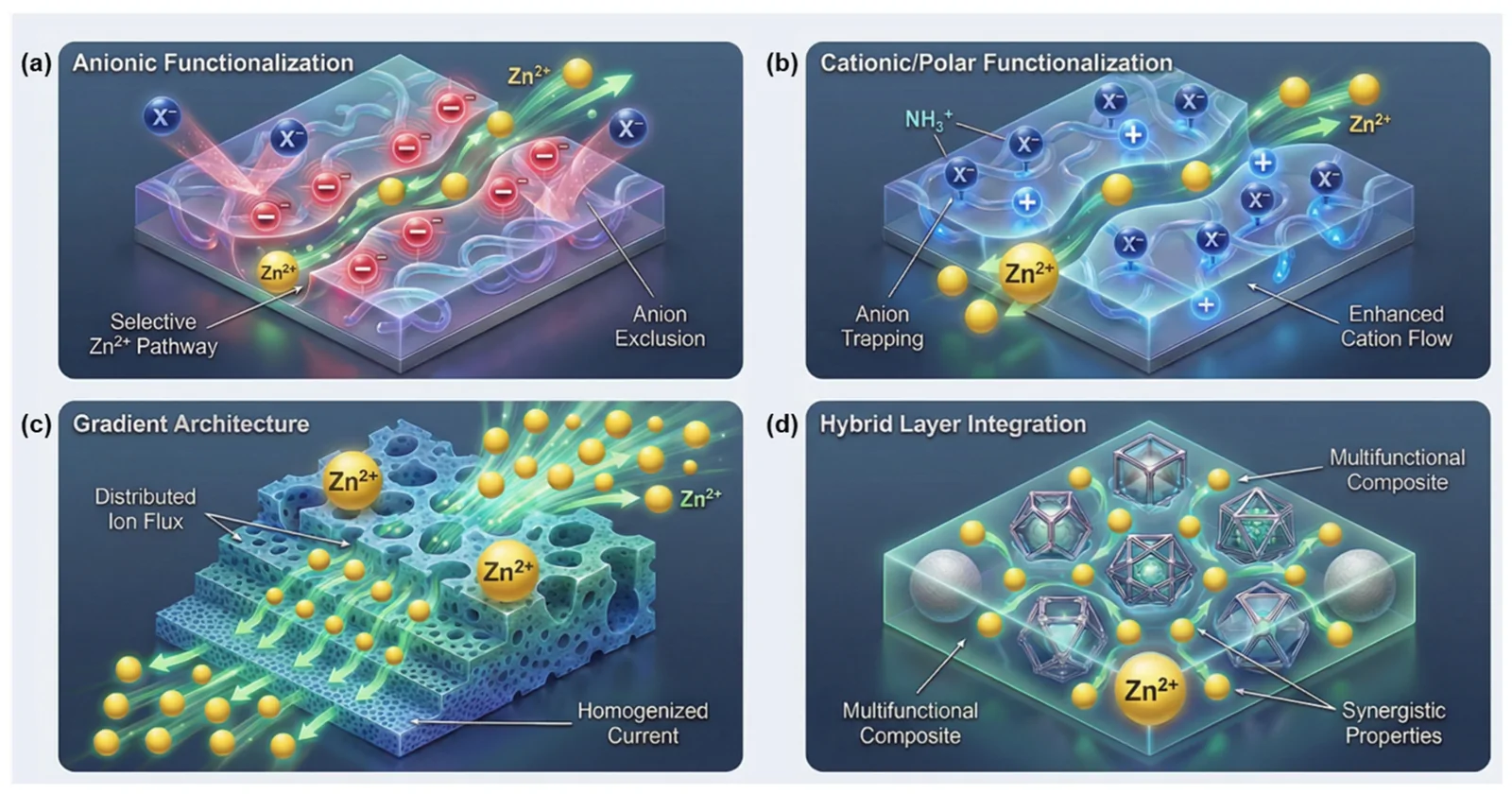
Design principles of separator architectures containing CP domains. Schematic illustration of four strategies for regulating Zn^2+^ transport and homogenizing interfacial ion flux while preserving electronic insulation. (**a**) Anionic functionalization, where fixed anionic groups, such as –COO^−^ and –SO_3_^−^, provide cation-selective sites that guide Zn^2+^ transport, regulate local solvation environments, and suppress anion crossover. (**b**) Cationic or polar functionalization, where amine-containing or polar sites immobilize anions, reduces space-charge accumulation and promotes a more uniform electric-field distribution near the Zn interface. (**c**) Gradient architecture, where spatial variation in pore size, functional-group density, or redox-state distribution redistributes interfacial polarization and supports more uniform Zn nucleation. (**d**) Hybrid layer integration, where CP-containing ion-regulating domains are combined with mechanically robust frameworks, such as MOF or ceramic domains, to balance selective ion transport, structural stability, and resistance to dendrite penetration.

Charge-selective surface engineering in separator architectures containing CP domains is governed by the sign, density, and spatial distribution of fixed-charge sites in the functional region (Figure 13). Negatively charged motifs, including carboxylate- and sulfonate-derived groups, can form cation-preferential microenvironments. The same motifs may facilitate partial desolvation and channel-guided Zn^2+^ transport. A quasi-solid polymer platform for Zn batteries showed that carboxylate-enriched microchannels increased the Zn^2+^ transference number and promoted more uniform metal deposition [69]. The result provides a design reference for separator-supported CP domains that contain fixed anionic sites. It should not be treated as direct evidence for CP-containing separators in Zn-ion hybrid capacitors.

A separator study in a monovalent-ion system can also clarify general permselectivity principles. Immobile anionic groups can suppress anion migration while maintaining cation pathways [125]. The mechanism provides a useful electrostatic analogy, although Zn^2+^ transport requires separate validation because divalent charge, hydration structure, and desolvation kinetics differ from those of monovalent ions. For Zn-ion hybrid capacitors, fixed anionic sites should therefore be evaluated using Zn^2+^ transference number, ionic resistance, leakage current, self-discharge behavior, and cycling stability.

Positively polarized functionalities and amine-enriched networks provide a second design lever. These functional groups can reduce anion mobility and moderate space-charge accumulation near metal surfaces. A representative amine-containing gel system increased cation transference by anion binding [126]. The result supports the mechanistic concept that anion immobilization can homogenize the local electrostatic driving force without reformulating the bulk electrolyte. For separator-related CP domains, the same principle must be combined with electronic discontinuity at the separator scale. CP-containing domains should regulate ion flux without forming continuous electron-transport pathways between electrodes.

Gradient design introduces additional control over ion transport and interfacial polarization. Gradients in pore size, functional-group density, CP content, or oxidation state can distribute ion flux over a larger length scale. The redistribution of ion flux may reduce localized overpotential near the metal interface. Asymmetric separator architectures, such as the Janus separator carrying an electroactive layer only on the Zn-facing side [127], provide a structural motif that can guide graded CP-containing separator designs in Zn^2+^ hybrid capacitors. Relevant implementation routes include control over polymerization depth, dopant diffusion, and spatial distribution of CP-enriched domains. Interphase-gradient concepts from high-voltage monovalent-cell studies provide additional guidance for spatially resolved interfacial chemistry [128]. Their use in Zn-ion hybrid capacitors should remain a design analogy unless Zn^2+^-based separator cells verify field homogenization, chemical stability, and charge retention.

Hybrid layer integration combines localized CP domains with rigid fillers or porous frameworks. The design target is to coordinate mechanical stability, adsorption of reactive species, and regulated ion transport in Zn^2+^-oriented separator architectures. A framework-based coating study in a sulfur system showed that polar frameworks can be integrated with redox-active polymer matrices to couple adsorption with transport regulation [129]. The specific shuttle chemistry is system-dependent, so the study should be cited as a materials-level design reference rather than direct Zn-ion hybrid capacitor evidence. For Zn systems, CP-containing hybrid layers should be assessed in terms of Zn^2+^ flux regulation, pore accessibility, dimensional stability, and suppression of electronic leakage.

Scalable fabrication is another required design axis. Vapor-phase polymerization and aqueous dispersion coating can form conformal CP-containing layers on porous separator substrates. Processing should create thin, localized, and electronically interrupted CP domains rather than continuous electronically conducting films. Key processing variables include CP loading, coating thickness, penetration depth, dopant distribution, and adhesion to the insulating separator scaffold. Practical separator design for Zn-ion hybrid capacitors therefore requires simultaneous control over ion selectivity, electronic insulation, swelling resistance, chemical durability, and manufacturing reproducibility.

Potential adverse effects deserve equally critical treatment. CP coatings add ionic resistance and can partially block pores, so a CP-modified separator may increase polarization at high current density even while homogenizing flux; coating thickness therefore trades off directly against rate capability, and porosity, tortuosity, electrolyte uptake, and Zn^2+^ transference should be reported together rather than separately. Repeated oxidation/reduction in CP domains can also induce swelling-driven cracking, delamination, dopant loss, or partial dissolution, any of which may re-open electronic pathways or alter pore connectivity during long-term cycling; post-cycling cross-sectional imaging combined with repeated leakage-current checks is the appropriate probe. Finally, attributing dendrite suppression to CP chemistry requires control separators with matched thickness, pore structure, wettability, and ionic resistance but without the CP phase; absent such controls, improvements cannot be separated from generic physical modification of the separator.

### 3.6. Summary of Separator-Level Functions

Separator architectures containing CP domains can provide functions beyond the passive spacing and ion-sieving roles of conventional porous polyolefin membranes in Zn-ion hybrid capacitors [130,131]. The separator itself must remain electronically insulating. Therefore, CP-containing regions should be described as spatially confined interfacial domains, partial functional layers, or components of insulating hybrid membranes, rather than as continuous conducting polymer separator films. Tailored fixed charges and coordination sites in CP-containing functional regions can impart ion-selective transport. The resulting cation-preferential pathways can promote a more uniform distribution of Zn^2+^ across the separator and near electrode interfaces [100,132]. Regulation of Zn^2+^ flux can reduce concentration gradients that drive dendritic growth and interfacial side reactions.

Localized redox-active CP domains may also contribute to charge buffering at separator-electrode interfaces [133]. The redox function should remain confined to local interfacial regions and should not create long-range electronic conduction across the separator. Controlled redox buffering may moderate local potential gradients and reduce polarization during high-rate operation. In contrast, excessive CP loading or electronically connected CP domains can increase leakage current, self-discharge, and short-circuit risk. Therefore, separator-level redox activity must be evaluated together with charge retention, leakage current, and long-term cycling stability.

CP-containing hybrid separator structures may also support mechanical stability at the separator scale. Insulating scaffolds, including polyolefin, cellulose-derived, glass-fiber, or inorganic-reinforced matrices, can preserve pore structure and dimensional stability [122]. CP-containing interfacial domains may improve electrolyte wetting, adhesion at the separator-electrode interface, and resistance to pore deformation. However, redox-state-dependent swelling or softening of CP-rich regions can alter pore accessibility and ion selectivity. Mechanical evaluation should therefore include dimensional stability, puncture resistance, pore retention, swelling ratio, and electrolyte uptake.

Thermal and chemical durability are also required for separator architectures containing CP domains. Surface modification can improve electrolyte affinity and delay thermal shrinkage when the insulating separator scaffold remains stable [134]. However, improved durability should not be assumed from the presence of CPs alone. The CP-containing region must resist overoxidation, dopant loss, hydrolysis, and dissolution in aqueous Zn^2+^ electrolytes. Thermal shrinkage tests, chemical aging, OCV hold measurements, and post-aging impedance analysis are needed to verify separator-level durability.

Electrostatic suppression of dendritic Zn growth arises from the combined regulation of ion selectivity and electric-field distribution. Fixed charges and polar coordination sites in CP-containing interfacial regions can redistribute Zn^2+^ flux near Zn metal anodes [100]. A more uniform distribution of Zn^2+^ can favor compact Zn deposition over filamentary growth. The same design must prevent electronic percolation across the separator thickness. Therefore, dendrite suppression should be discussed together with electronic insulation, self-discharge behavior, and Zn plating-stripping stability.

In summary, separator architectures containing CP domains should be viewed as ion-regulating and interface-stabilizing separator systems with preserved electronic insulation. Their functions include Zn^2+^ flux regulation, local charge buffering, wetting retention, field homogenization, resistance to pore deformation, and durability under thermal or chemical stress [135]. The central design requirement is to combine these functions without forming continuous electronic pathways between electrodes. When integrated with electrolyte-level and electrode-level CP functions, separator-level CP domains can contribute to Zn-ion hybrid capacitors through controlled interfacial chemistry, safer ion transport, and scalable processing routes. However, device-level claims require direct evidence from Zn-ion hybrid capacitor cells, including leakage current, self-discharge rate, Zn^2+^ transport metrics, cycling stability, and post-cycling separator analysis [122].

## 4. Design Framework for Zn Hybrid Capacitors Containing CPs

Zn-ion and alkali-ion hybrid capacitors share a device-level objective: rapid and stable charge transport across electrolyte, separator, and electrode interfaces during prolonged cycling. However, charge-carrier valence, solvation structure, and interfacial reactivity impose different physicochemical constraints on each system. Zn-ion hybrid capacitors are particularly distinct because Zn^2+^ combines divalent charge with strong hydration and metal-plating chemistry. The design of CP-containing components must therefore address Zn^2+^ transport, desolvation, water-driven side reactions, dendritic deposition, and electronic leakage.

Section 2 and Section 3 summarized electrolyte-level and separator-level mechanisms involving CPs. In electrolytes, CP-containing domains can regulate ion coordination, local redox buffering, and interfacial charge distribution. In separator architectures, CP domains should remain spatially confined and electronically interrupted. The separator must preserve electronic insulation while allowing Zn^2+^ transport. This section formulates a Zn-centered design framework for hybrid capacitors containing CPs; studies of Li^+^, Na^+^, and K^+^ are referenced only when comparative data clarify mechanistic features relevant to Zn^2+^ systems.

### 4.1. Distinct Electrochemical Environments

**Figure 14:**
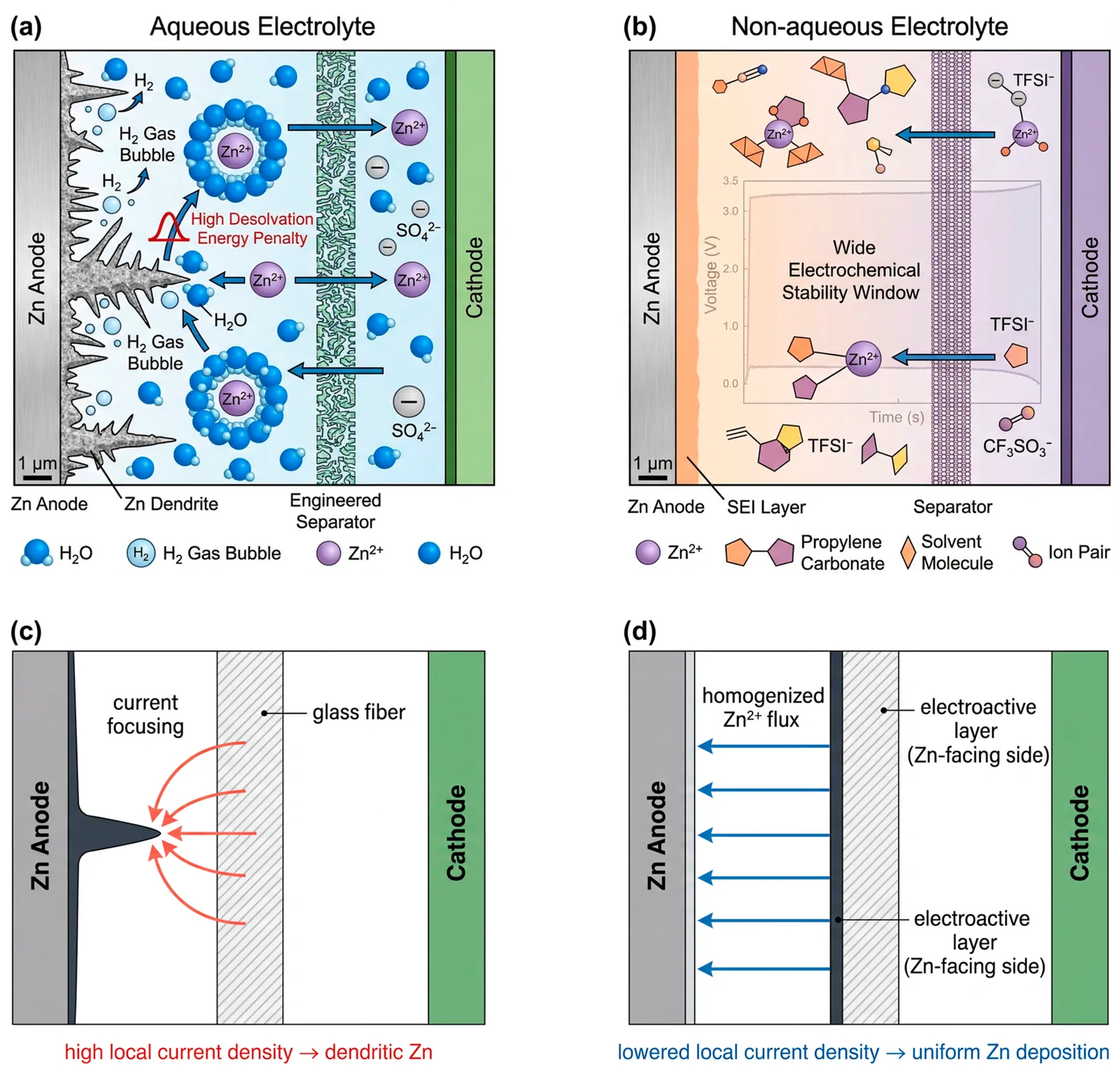
Electrolyte-dependent electrochemical environments and design challenges of Zn-ion hybrid capacitors. (**a**) In aqueous electrolytes, hydrated Zn^2+^ transport, desolvation at the Zn interface, hydrogen evolution, corrosion, by-product formation, and nonuniform ion flux can jointly promote unstable Zn deposition. Therefore, electrolyte formulation, separator architecture, and interfacial regulation should be designed to control water activity, Zn^2+^ flux, and local electric-field distribution. (**b**) Nonaqueous electrolytes can provide a wider electrochemical stability window, although ion pairing, sluggish Zn^2+^ transport, solvent and salt decomposition, and solid electrolyte interphase (SEI) instability introduce different constraints. The design focus therefore shifts from hydration- and water-activity-controlled interfacial kinetics in aqueous media to solvation-, ion-pairing-, and SEI-controlled stability in nonaqueous formulations. (**c**,**d**) Schematic comparison of separator-side boundary-condition control in aqueous Zn systems: (**c**) a conventional separator permits current focusing at surface protrusions and dendritic Zn growth, whereas (**d**) a Janus-type separator carrying an electroactive layer on the Zn-facing side lowers the local current density, homogenizes Zn^2+^ flux, and promotes uniform Zn deposition. The schematics were redrawn based on the Janus separator concept reported by Li et al. [127].

Zn-ion hybrid capacitors have been reported mainly in aqueous electrolytes, where Zn^2+^ migrates as a hydrated divalent carrier. Strong hydration increases the energetic and kinetic penalty of interfacial desolvation and charge transfer. High-rate operation can further amplify concentration polarization near the Zn metal interface. Electrolyte and separator designs for aqueous Zn systems must therefore suppress water-coupled parasitic reactions and interfacial heterogeneity. Key failure modes include hydrogen evolution, corrosion, nonuniform Zn deposition, and dendritic protrusion formation (Figure 14a) [136].

Zn-ion hybrid capacitors have also been reported in nonaqueous electrolytes [137]. In these systems, Zn salts are dissolved in organic solvents to access different balances among voltage stability, ion pairing, and transport kinetics. Nonaqueous Zn electrolytes require control over solvent decomposition, salt dissociation, and interphase formation at negative electrodes. The multivalent character of Zn^2+^ and its relatively slow desolvation remain relevant. As a result, Zn-ion hybrid capacitors span a continuum from hydration-controlled interfacial kinetics in aqueous media to organic-solvation-controlled stability limits in nonaqueous liquid or gel formulations (Figure 14b).

Li et al. [127] demonstrated a representative separator-side strategy for boundary-condition control using a Janus separator. The Janus separator was fabricated by growing a vertical graphene carpet on one side of a commercial glass-fiber separator. Figure 14c,d schematically compare a conventional glass-fiber separator with the Janus architecture. The Janus architecture was designed to lower local current density and homogenize ion distribution near the Zn anode interface. The study shows that separator engineering in aqueous Zn systems often targets local ion-flux regulation and interfacial electrostatic uniformity, rather than only extending the voltage window. Notably, this architecture also provides one of the few direct hybrid capacitor validations to date: an activated-carbon‖Zn cell with the Janus separator retained 93% of its capacity over 5000 cycles at 5 A g^−1^ [127].

Comparative data from Li^+^, Na^+^, and K^+^ hybrid capacitors should be used as mechanistic references for polymer-rich nonaqueous Zn electrolytes. Conductivity, ion pairing, and durability trends reported for alkali-ion gels and polymer electrolytes can help identify design variables for CP-containing Zn electrolytes [138,139]. The primary focus should remain on Zn-based architectures. Alkali-ion data should not replace direct evidence from Zn-ion hybrid capacitor cells because Zn^2+^ has different charge density, coordination behavior, and desolvation kinetics.

Aqueous Zn systems provide the main implementation platform for the design framework developed in the following sections. Nonaqueous and gel-based Zn electrolytes extend the same logic toward wider voltage operation. Alkali-ion analogs can support interpretation when the comparison is limited to general polymer–electrolyte physics. Section 4.2 examines how CP-containing domains influence ion transport and interfacial stability across Zn environments. Section 4.3 then organizes the discussion using a valence–hydration–interface coupling framework [136,139].

### 4.2. Mechanistic Roles of CPs in Zn-Based Hybrid Capacitors

The redox activity, fixed-charge chemistry, and strain-accommodation behavior of CPs can contribute to electrolyte and separator design in hybrid capacitors. However, the mechanistic role of CPs is determined by the electrochemical environment and by the location of the CP-containing domain. In Zn-ion hybrid capacitors, CPs should therefore be evaluated through Zn^2+^ transport, hydration structure, interfacial reactivity, and electronic-leakage risk, rather than through the general properties of CPs alone.

In aqueous Zn electrolytes, Zn^2+^ migrates as a strongly hydrated divalent carrier. The large hydration shell and desolvation penalty of Zn^2+^ slow interfacial charge transfer and increase concentration polarization under high current density. The Zn metal surface also undergoes water-driven reactions, including hydrogen evolution and corrosion. Nonuniform current distribution can further promote morphological instability and dendritic Zn growth. Therefore, CP-containing electrolyte domains and separator-facing interfacial domains should be designed to regulate Zn^2+^ flux, local charge accumulation, and water-related side reactions at the Zn interface.

**Figure 15:**
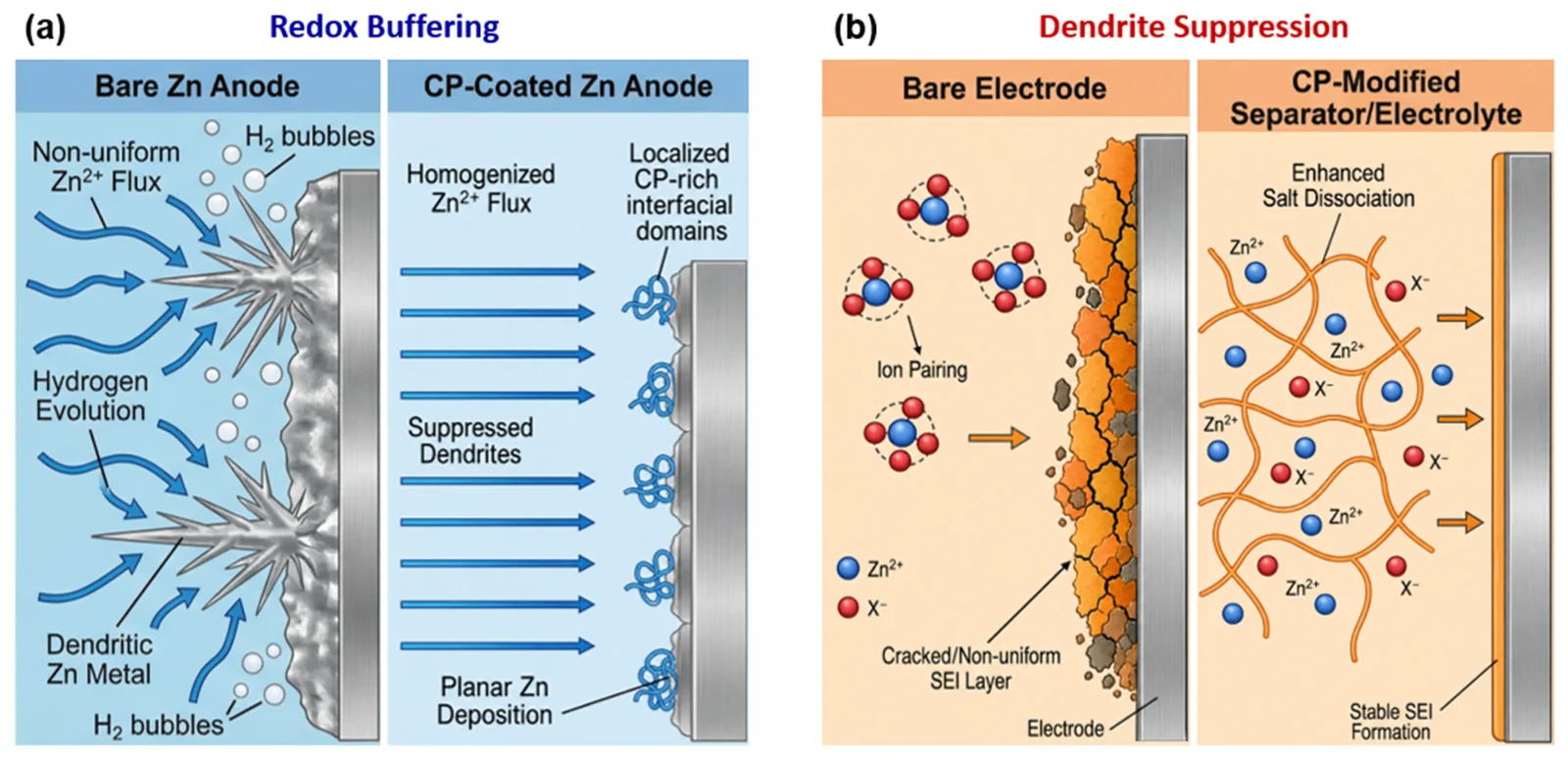
Environment-dependent mechanistic roles of CP-containing interfacial domains in Zn-based hybrid capacitors. (**a**) In aqueous electrolytes, nonuniform current distribution at a bare Zn anode can promote dendritic growth, hydrogen evolution, corrosion, and by-product formation. CP-containing interfacial domains can regulate Zn^2+^ flux through fixed-charge interactions and local electric-field redistribution, while compliant polymer regions can accommodate plating- and stripping-induced stress. These effects support more uniform Zn deposition and improved cycling stability when electronic insulation is preserved. (**b**) In nonaqueous electrolytes, ion pairing, sluggish Zn^2+^ transport, solvent or salt decomposition, and unstable SEI formation become dominant constraints. CP-containing electrolyte or separator-related architectures can tune local polarity, promote salt dissociation, and assist formation of a more uniform interphase, provided that long-range electronic percolation, leakage current, and self-discharge are controlled.

CP-containing electrolyte domains may contribute fixed-charge sites and redox-buffering centers that moderate local potential gradients. Separator-supported CP domains can provide polar coordination environments and localized charge compensation near the separator-electrode interface. These functions should remain spatially confined. They should not create continuous pathways of electronic conduction across the separator or electrolyte region. Controlled redistribution of Zn^2+^ flux and interfacial charge can reduce local overpotential and suppress electric-field amplification at surface protrusions. As a result, Zn deposition may proceed with a more uniform morphology, as schematically shown in Figure 15a [2].

Mechanical effects should also be described in terms of specific deformation processes. CP-containing interfacial domains may accommodate local strain generated by uneven Zn plating and stripping. Improved retention of contact at the electrode–electrolyte or electrode–separator interface can reduce crack formation, delamination, and local thinning of separator regions. For separator-related structures, the mechanical benefit is meaningful only when pore accessibility and electronic insulation are preserved. Excessive swelling or electronic connectivity of CP-rich domains can increase ionic resistance, leakage current, or self-discharge.

Structured separators, including Janus separators and separators with graded porosity, provide additional routes for field homogenization and ion-flux redistribution. When CP-containing domains are integrated into structured separators, the design target is spatial separation of functions. Ion guidance, local redox buffering, electrolyte wetting, and strain accommodation should occur without long-range electron transport across the separator. Therefore, CP-rich regions should be localized, electronically interrupted, and supported by an insulating scaffold.

In nonaqueous or gel polymer electrolytes containing Zn salts, the dominant limitations differ from those in aqueous Zn systems. Solvent decomposition, salt dissociation, ion pairing, and interphase formation become more important. The multivalent character of Zn^2+^ and its desolvation barrier remain relevant. Studies on alkali-ion hybrid capacitors, especially K-based systems, have shown that CP-containing separators or electrolyte matrices can influence dielectric response, salt dissociation, and interphase composition [138,139]. In the Zn-focused framework of the present review, alkali-ion results are used as mechanistic analogs. They do not substitute for direct evidence obtained from Zn-ion hybrid capacitor cells.

Alkali-ion precedents can nevertheless guide hypotheses for nonaqueous Zn electrolytes. Polymer-rich environments may be tuned to improve salt dissociation, reduce ion pairing, and stabilize interfacial chemistry. For Zn salts, however, CP chemistry must be adjusted to account for Zn^2+^ charge density, coordination strength, and slower desolvation. Figure 15b summarizes the transition from hydration-controlled Zn chemistry in aqueous media to solvation- and interphase-controlled constraints in nonaqueous or gel Zn electrolytes.

Taken together, CPs operate as environment-dependent interfacial regulators in Zn-based hybrid capacitors. In aqueous Zn electrolytes, fixed-charge sites and redox-active backbones can regulate Zn^2+^ flux, local pH variation, and interfacial charge distribution. In nonaqueous or gel Zn electrolytes, related structural motifs may tune dielectric response, salt dissociation, and interphase stability. Across both environments, CP-containing domains must be designed to support ion transport and local charge regulation while suppressing electronic leakage, self-discharge, uncontrolled swelling, and loss of separator insulation.

### 4.3. Valence–Hydration–Interface Coupling Framework

**Figure 16:**
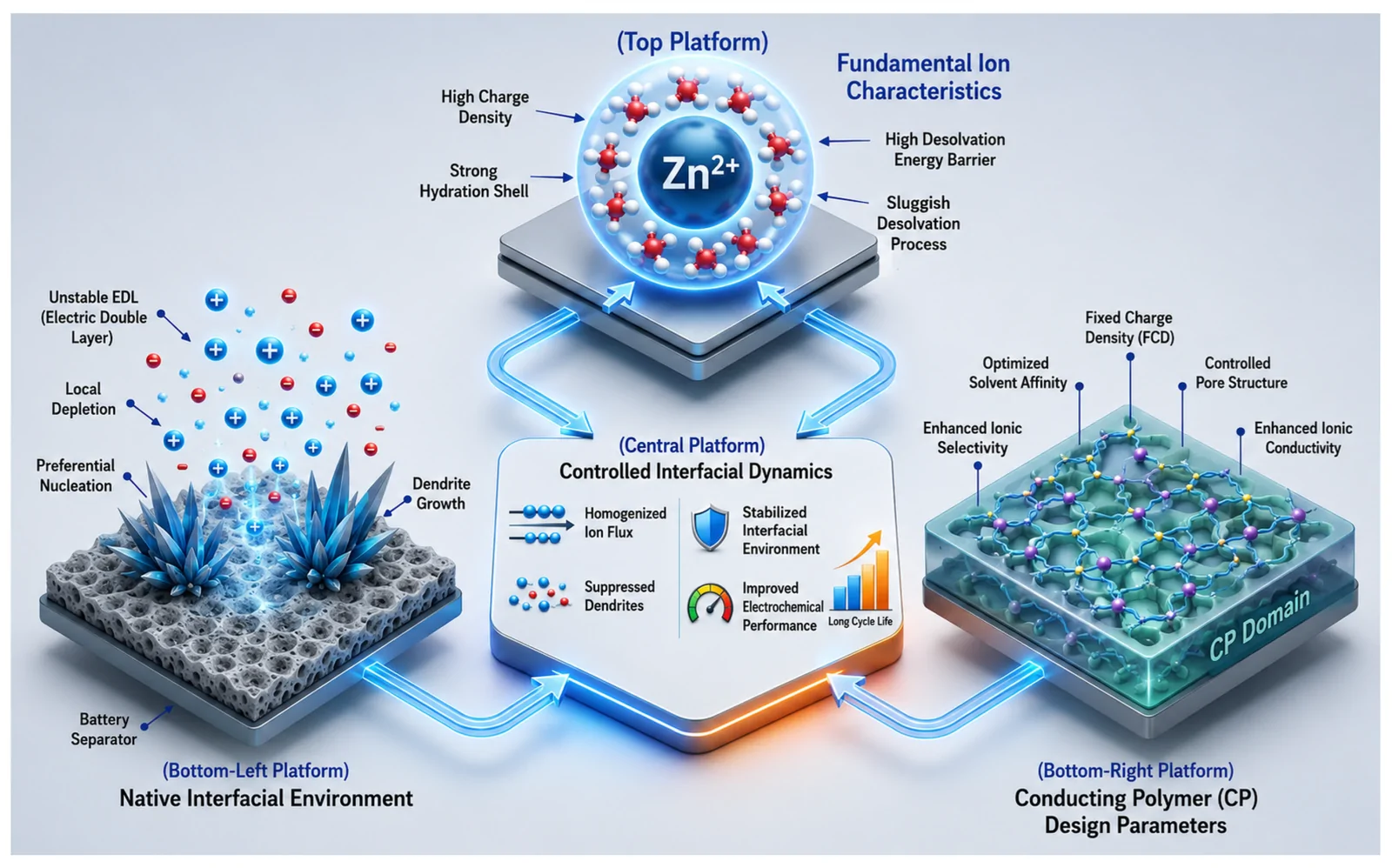
Valence, hydration, and interface coupling framework for separator architectures containing CP domains in Zn-ion hybrid capacitors. The schematic links intrinsic Zn^2+^ properties, native interfacial instability, CP-domain design parameters, and controlled interfacial dynamics. The top platform describes the fundamental ion characteristics of Zn^2+^, including high charge density, a strongly hydrated coordination shell, and the associated desolvation barrier at the electrode interface. The bottom-left platform illustrates the native interfacial environment in conventional separator systems, where nonuniform electric-field distribution, local ion depletion, preferential nucleation, and dendritic growth can occur. The bottom-right platform summarizes CP-domain design parameters, including fixed-charge density, solvent affinity, pore structure, ionic selectivity, and ionic conductivity. Spatially confined CP domains or interlayers are designed to guide Zn^2+^ transport while preserving electronic insulation. The central platform summarizes the expected interfacial outcomes, including homogenized ion flux, suppressed dendrite formation, stabilized interfacial chemistry, and improved electrochemical performance.

A design framework for separator architectures containing CP domains in Zn-ion hybrid capacitors can be formulated by considering three coupled variables: ion valence, hydration or solvation structure, and interfacial architecture. Zn^2+^ has a higher charge density than monovalent alkali ions. The divalent charge of Zn^2+^ produces stronger electrostatic perturbation near metal–electrolyte and separator–electrolyte interfaces. The hydration shell of Zn^2+^ also increases the effective ion size and the desolvation barrier. As a result, Zn^2+^ transport is closely linked to water activity, local pH variation, and interfacial charge distribution (Figure 16) [136].

These features determine how separator morphology and surface chemistry influence Zn deposition. Small changes in pore geometry, fixed-charge density, or surface roughness can alter nucleation overpotential, local pH, and dendrite initiation. CP-containing separator domains can adjust the boundary conditions encountered by Zn^2+^ before it reaches the metal surface. Fixed anionic motifs can regulate Zn^2+^ transference, local water activity, and electrostatic screening [2,127]. Localized redox-active domains may buffer transient overpotentials near separator-electrode interfaces. Mechanically compliant interfacial regions may also accommodate strain generated by uneven Zn plating and stripping.

For separator applications, CP-containing domains must remain spatially confined and electronically interrupted. The function of CP domains should be ion-flux regulation, local charge buffering, and interfacial stabilization. The function should not be long-range electronic conduction across the separator. Continuous electronic pathways can increase leakage current, self-discharge, and short-circuit risk. Therefore, valence–hydration–interface coupling must be considered together with electronic percolation control.

In nonaqueous or gel polymer media containing Zn salts, the dominant constraints shift from hydration and water activity to ion pairing, solvent stability, and interphase formation. CP-containing domains may influence dielectric response, salt dissociation, and interphase chemistry under these conditions. Studies on K-based and other alkali-ion hybrid capacitors provide mechanistic references for these effects [138,139]. However, alkali-ion data should not be treated as direct evidence for Zn-ion hybrid capacitors. Zn^2+^ requires separate validation because divalent charge, coordination strength, and desolvation behavior differ from those of Li^+^, Na^+^, and K^+^.

The valence–hydration–interface framework suggests three design parameters for separator architectures containing CP domains. First, fixed-charge density and spatial distribution should provide Zn^2+^ selectivity without excessive double-layer crowding or uncontrolled pH gradients. Second, the hydration or solvation affinity of CP backbones, dopants, and side groups should balance Zn^2+^ mobility with control of water activity in aqueous electrolytes or solvent stability in nonaqueous electrolytes. Third, the length scale of CP-mediated interfacial modulation should match the length scales of electric-double-layer structure, ion depletion, and morphology instability. Proper matching can dampen local perturbations rather than amplify them.

This framework links electrolyte-level and separator-level functions in Zn-ion hybrid capacitors. It also provides a basis for comparing aqueous Zn platforms with nonaqueous Zn systems. Alkali-ion studies can support interpretation when the comparison is limited to general principles of ion coordination, interphase formation, or polymer-mediated transport [138,139]. Direct claims regarding Zn-ion hybrid capacitors should rely on Zn^2+^-based measurements, including Zn^2+^ transference number, ionic resistance, leakage current, self-discharge rate, Zn deposition morphology, and cycling stability.

Table 2 consolidates the representative CP-containing and benchmark systems discussed in Section 2 and Section 3, together with their reported performance indicators, the class of evidence each provides for Zn-ion hybrid capacitors, and whether the corresponding design concept is demonstrated, partially demonstrated, or proposed. Two features stand out: only one of the flagship systems—the electroactive Janus separator of ref [127]—has so far been validated in a complete Zn-ion hybrid capacitor cell, and Zn^2+^ transference numbers are almost never reported. These two gaps define the most direct experimental priorities for the field.

**Table 2:** Representative CP-containing and benchmark systems discussed in this review, with reported performance indicators, evidence class, and demonstration status.

System [Ref]	Role	Medium	Key Reported Indicators	Evidence Class	Status
PEDOT:PSS/MoS_2_ (PEPM) coating on Zn [104]	Anode-side interfacial layer	2 M ZnSO_4_ (aq)	Zn‖Zn stability > 2100 h at 1 mA cm^−2^; dendrite-free plating	Zn battery	Demonstrated
GF/PANI-600 separator [106]	Zn-facing separator modification	2 M ZnSO_4_ (aq)	Zn‖Zn ≈3000 h; Zn‖MnO_2_ 89.5% retention after 300 cycles (0.5 A g^−1^)	Zn battery	Demonstrated
Agarose/Na-alginate bio-GPEs [45]	Aqueous GPE	2 M ZnSO_4_ in gel	Dendrite/by-product suppression; ≥liquid-cell capacity at high loading	Zn battery	Demonstrated
Free-standing 2D/3D PEDOT films (ITIES) [115]	Membrane concept	Aq./org. interface	Tunable growth mode; film thickness ≈50–850 nm	Materials-level	Partially demonstrated
Capillary-cell Li electrodeposition [117]	Mechanistic model of dendrite onset	1 M LiPF_6_, EC/DMC	Mossy → dendritic transition at Sand’s time (2.61 mA cm^−2^)	Li analog	Demonstrated (mechanism)
External AC/DC-field diffusion enhancement [118]	Field-controlled deposition	Li metal cell	≈5× lifespan; compact deposition without material change	Li analog	Demonstrated (mechanism)
PE-SiO_2_@PDA separator [120]	Thermal-durability benchmark	Li-ion cell	≈0% shrinkage to 230 °C; stable OCV at 170 °C; 93.9% after 200 cycles	Li benchmark	Demonstrated
DMAET-PE/silica-PE separators [121]	Surface-modified separator	Li-ion cell	Reduced shrinkage after 140 °C/1 h; OCV-stable	Li benchmark	Demonstrated
Aqueous-Zn separator criteria [122]	Chemical-durability benchmarks	1 M ZnSO_4_ (aq)	pH 3–14 tolerance; <1% mass loss after 30 d	Zn battery (criteria)	Benchmark
Conformal PEDOT on LiCoO_2_/Ni-rich cathodes [123,124]	Electrode-interface analog	Organic Li-ion cell	Stable 3.0–4.5 V (LiCoO_2_); 2.8–4.6 V, 200 cycles (Ni-rich)	Li analog	Demonstrated
VG@GF Janus separator [127]	Zn-facing electroactive separator	Aq. Zn	Zn‖Zn > 300 h (0.5 mA cm^−2^); AC‖Zn hybrid capacitor: 93% retention over 5000 cycles (5 A g^−1^)	Zn-ion hybrid capacitor	Demonstrated
Redox-active CP electrolyte additives (Section 2.3.3)	Electrolyte concept	Aq. Zn (proposed)	n.r.	—	Proposed

Evidence class denotes the system in which the listed indicators were obtained: “Zn-ion hybrid capacitor”, complete hybrid-capacitor cells; “Zn battery”, Zn-based full or symmetric cells; “Li analogue”/“Li benchmark”, lithium-based cells providing mechanistic or durability reference; “Materials-level”, film-level characterization without cell-level validation. Status summarizes the demonstration level of each concept: “Demonstrated”, supported by cell-level data in the listed system; “(mechanism)”, the demonstration concerns a transport or deposition mechanism rather than a component technology; “Partially demonstrated”, supported only by materials-level evidence; “Proposed”, a design concept not yet tested experimentally; “Benchmark”, acceptance criteria rather than a device demonstration. n.r., not reported in the cited study; VG@GF, vertical-graphene-coated glass-fiber separator; AC, activated carbon.

## 5. Outlook and Perspectives

CPs have moved beyond their conventional use as electronically conducting additives and are now being considered as localized polymer domains that may regulate ion transport, local charge compensation, and interfacial stability in energy-storage systems. In Zn-ion hybrid capacitors, CP-containing electrolytes and separator-related architectures are relevant because Zn^2+^ transport is coupled with hydration, desolvation, water-related side reactions, and dendritic Zn deposition. The electrochemical role of CPs should therefore be defined by their spatial position, redox state, and electronic connectivity in the cell. CP-containing domains may support local ion regulation and redox buffering, whereas electronically continuous pathways may induce leakage current, self-discharge, or short-circuit failure. Alkali-ion systems can provide mechanistic references, although conclusions from these systems should be transferred to Zn-ion hybrid capacitors only with Zn-specific validation.

CP-containing electrolytes and separator-related architectures are more appropriately viewed as engineered interfacial systems than as materials designed only to maximize bulk conductivity. The central design objective is to regulate ion activity, electric-field distribution, redox-state evolution, and charge retention at electrolyte-separator-electrode contacts during cycling. For separator applications, CP domains should remain spatially confined and electronically interrupted. The separator scaffold must preserve electronic insulation, pore accessibility, dimensional stability, and chemical resistance.

Comparison between Zn-ion and alkali-ion platforms indicates that CP function is strongly dependent on electrolyte chemistry and interfacial reaction pathways. In aqueous Zn-ion systems, CP design should prioritize Zn^2+^ transport, regulation of water activity, suppression of hydrogen evolution, and uniform Zn deposition. Charge-selective motifs and controlled redox states may contribute to Zn^2+^ flux regulation and interfacial charge redistribution. In nonaqueous or gel-based Zn systems, CP-containing domains may instead influence dielectric response, ion pairing, solvent decomposition, and interphase composition. Findings from Li-, Na-, and K-ion systems can guide mechanistic interpretation, whereas direct claims for Zn-ion hybrid capacitors require validation in Zn^2+^-based cells.

**Figure 17:**
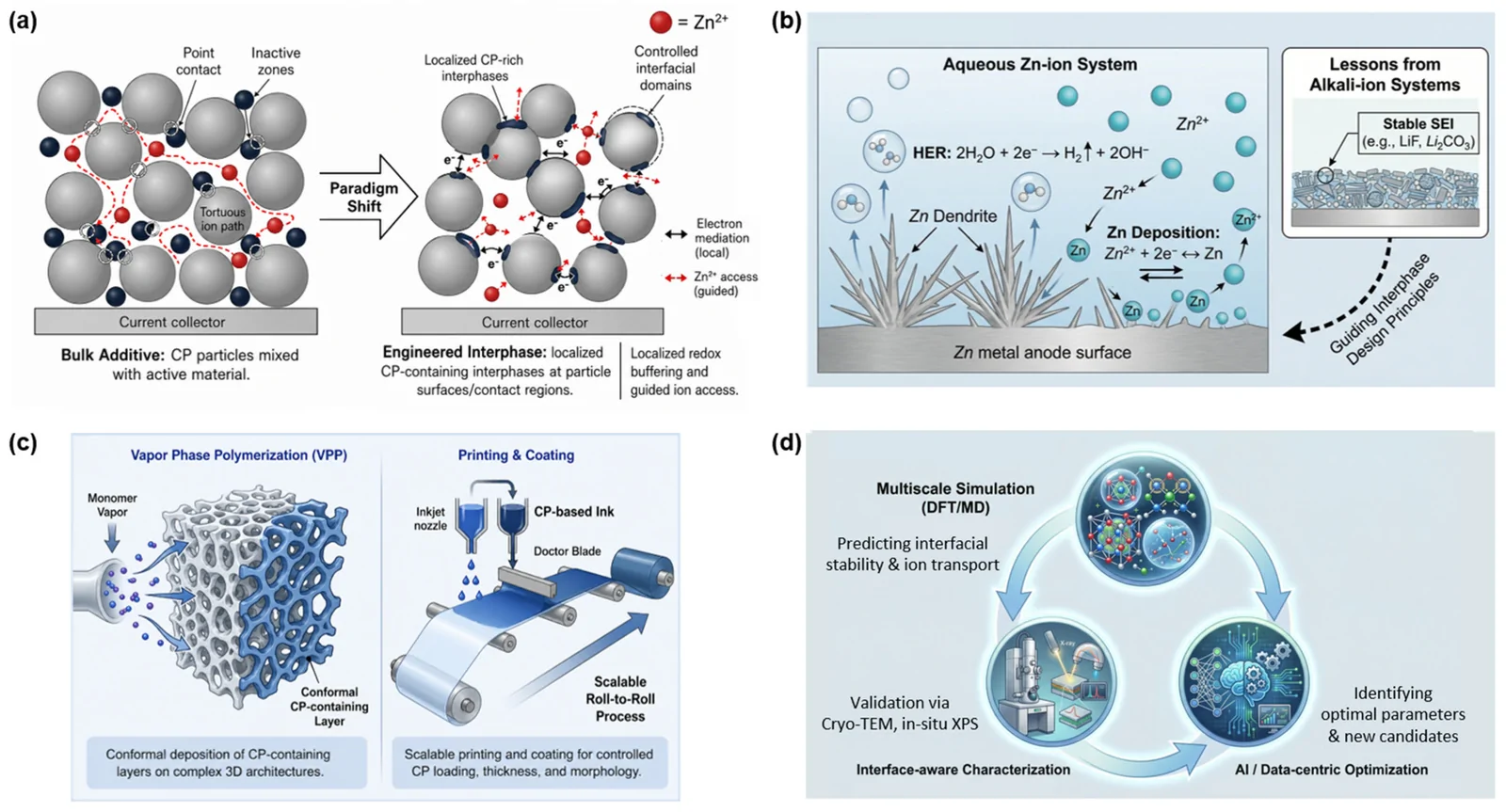
Outlook and perspectives for CP-containing interphases in Zn-ion systems. (**a**) Conceptual transition from dispersed CP additives to engineered CP-containing interphases with controlled spatial distribution, interfacial contact, and ion-accessible pathways. The interphase-oriented design aims to improve local charge buffering and ion transport while limiting uncontrolled electronic connectivity. (**b**) Strategic priorities for aqueous Zn-ion systems include suppression of hydrogen evolution, corrosion, by-product formation, and dendritic Zn growth. Mechanistic concepts developed in nonaqueous alkali-ion systems can guide interphase design, although Zn-specific validation is required because aqueous Zn chemistry involves hydration, desolvation, and parasitic water-related reactions. (**c**) Scalable manufacturing routes for CP-containing interfacial architectures. Vapor-phase polymerization, printing, coating, and roll-to-roll processing can support controlled CP loading, thickness, morphology, and substrate compatibility for practical separator, electrolyte, or electrode-interface designs. (**d**) Accelerated discovery loop integrating multiscale simulations, interface-sensitive characterization, and data-driven optimization. DFT and MD can screen interfacial stability and ion transport descriptors, while cryo-TEM, in situ or operando spectroscopy, and XPS-based analyses can validate interphase structure and chemistry. Data-centric optimization can connect synthesis variables, interfacial descriptors, and electrochemical metrics to guide future CP-containing Zn-ion systems.

Future studies should place greater emphasis on quantitative validation of CP function under device-relevant conditions (Figure 17). CP-containing electrolytes should be evaluated using transport and stability metrics, including ionic conductivity, Zn^2+^ transference number, Coulombic efficiency, voltage window, leakage current, self-discharge rate, swelling ratio, and cycling retention. Separator-related architectures containing CP domains should be tested for electronic insulation at the device level. Thermal shrinkage, puncture resistance, pore retention, electrolyte uptake, and post-cycling morphology should be reported together with electrochemical performance. Redox-active CP additives and swollen CP domains also require careful assessment of mediator retention, redox reversibility, and shuttle-induced self-discharge.

The scalable fabrication of CP-containing domains remains an important requirement for reliable implementation. Future processing routes should control the thickness, dopant distribution, and interfacial placement of CP-containing domains. Water-based coating, printing, oxidative polymerization, vapor-phase polymerization, and electrodeposition provide relevant routes for preparing CP-containing electrolyte and separator-related architectures. The practical value of each route will depend on whether CP-containing domains can be positioned without uncontrolled penetration through separator pores or long-range electronic percolation. Reproducible manufacturing will require control over CP loading, coating uniformity, adhesion to insulating scaffolds, and chemical stability under aqueous Zn-ion conditions.

Further progress will require closer coupling between mechanistic modeling, operando characterization, and data-guided optimization. Multiscale modeling can help connect Zn^2+^ coordination, hydration structure, dopant chemistry, and interfacial potential distribution. Operando Raman spectroscopy, FTIR spectroscopy, EQCM, impedance analysis, and imaging methods are needed to verify ion-coupled redox processes and morphology evolution. Data-guided optimization can then relate polymer structure, dopant selection, electrolyte composition, and separator architecture to device-level metrics. Computational and data-driven methods should support, rather than replace, Zn-specific electrochemical validation.

Priority open questions. Framed by the evidence classification above, five questions take precedence before CP-containing electrolytes and separators can be considered practically viable for Zn-ion hybrid capacitors: (1) direct validation—do CP-containing electrolytes or separators improve complete Zn-ion hybrid capacitor cells at realistic mass loadings and electrolyte-to-capacity ratios, benchmarked against CP-free controls of matched physical properties? (2) Zn^2+^ selectivity—what are the Zn^2+^ transference numbers of the leading CP-containing systems, and do fixed anionic sites enhance or immobilize Zn^2+^ at the densities actually used? (3) Electronic boundaries—what CP loadings and architectures keep leakage current and self-discharge below application thresholds throughout cycling, including after repeated CP redox? (4) Redox-active electrolytes—does a beneficial concentration window exist in which localized buffering outweighs shuttle losses? (5) Durability—do CP domains retain electronic discontinuity, adhesion, and dopant inventory over thousands of cycles in hydrated Zn^2+^ electrolytes? The concepts in this review should be read against this list: architectures marked demonstrated in Table 2 rest on Zn-cell, Li-cell, or—in one case [127]—direct hybrid capacitor data, whereas those marked proposed, including redox-active electrolytes with controlled electronic coupling, remain design hypotheses awaiting device-level tests.

Overall, the significance of CPs in Zn-ion hybrid capacitors is expected to depend on interfacial function under well-defined architectural constraints. CP-containing electrolytes and separator-related architectures may connect ion valence, solvation structure, and interfacial response in a unified design framework. Their practical value will depend on balancing Zn^2+^ transport and local redox regulation with suppression of electronic leakage, self-discharge, swelling, and chemical degradation. Future progress should therefore move from qualitative demonstrations to quantitative benchmarking and device-specific validation, beginning with the five questions above. A cautious comparison with alkali-ion systems will help clarify which CP-based strategies are transferable to Zn-ion hybrid capacitors and which mechanisms require direct Zn-specific evidence.

## Figures and Tables

**Table 1:** Cross-cutting design constraints and validation criteria for CP-containing electrolyte and separator-related architectures.

Design Constraint	Electrolyte	Separator	Risk	Validation	Interpretation
Long-range electronic connectivity	Excessive mobility or connectivity of redox-active CP chains can mediate charge transfer across the electrolyte phase.	Connected CP domains can form through-plane electron pathways across separator scaffolds.	Leakage current, self-discharge, redox shuttle, or short circuit.	Electronic conductivity, leakage current, open-circuit voltage decay, self-discharge, rest-period cycling.	CP domains should be described as electronically limited, spatially confined, or electronically interrupted.
CP loading and pore accessibility	High CP content can increase viscosity, reduce ion mobility, and alter electrolyte wetting.	Excessive CP loading can block pores, reduce electrolyte uptake, and increase ionic resistance.	Increased polarization, reduced rate capability, pore obstruction, and unstable ion flux.	Ionic conductivity, Zn^2+^ transference number, viscosity, electrolyte uptake, porosity, electrochemical impedance spectroscopy (EIS), rate performance.	CP-containing regions should be discussed as regulated functional domains rather than continuous CP-rich phases.
Redox activity and charge retention	Mobile redox-active CP species can induce parasitic charge exchange or shuttle behavior.	Redox-active CP domains can promote self-discharge when electronic isolation is insufficient.	Coulombic inefficiency, capacity fading, and charge loss during rest.	CV, EQCM, leakage current, Coulombic efficiency, self-discharge, cycling with rest intervals.	Redox activity should be framed as local charge buffering only when reversibility and retention are verified.
Dimensional and chemical stability	Solvent uptake, dopant migration, or pH-dependent degradation can alter ion pathways.	Swollen or degraded CP domains can close pores, deform scaffolds, or lose interfacial function.	Pore closure, dimensional drift, increased impedance, and shortened cycle life.	Swelling ratio, thickness change, mechanical testing, X-ray photoelectron spectroscopy (XPS), FTIR, electrolyte aging, post-cycling morphology.	Stability should be linked to measurable retention of dimensions, pore structure, and chemical functionality.
Ion selectivity and coordination	Functional groups can tune Zn^2+^ solvation, ion pairing, and charge compensation.	Fixed charges and polar sites can regulate Zn^2+^ flux and suppress anion crossover.	Poor Zn^2+^ transport, concentration polarization, nonuniform deposition, or reduced selectivity.	Zn^2+^ transference number, ion-pair analysis, Raman, FTIR, nuclear magnetic resonance (NMR), concentration-gradient analysis, Zn morphology.	Solvation or desolvation regulation should be claimed only with direct spectroscopic, computational, or transport evidence.

## Data Availability

No new data were created or analyzed in this study. Data sharing is not applicable to this article.

## Abbreviations

The following abbreviations are used in this manuscript:

2D	two-dimensional
3D	three-dimensional
AFM	atomic force microscopy
CP	conducting polymer
CV	cyclic voltammetry
DFT	density functional theory
DMAET	2-(dimethylamino)ethanethiol
DPSCA	double-potential step chronoamperometry
EC/DMC	ethylene carbonate/dimethyl carbonate
EIS	electrochemical impedance spectroscopy
EQCM	electrochemical quartz crystal microbalance
FTIR	Fourier transform infrared
GF	glass fiber
GPE	gel polymer electrolyte
HER	hydrogen evolution reaction
ITIES	interface between two immiscible electrolyte solutions
MD	molecular dynamics
MOF	metal–organic framework
NMR	nuclear magnetic resonance
OCV	open-circuit voltage
PANI	polyaniline
PDA	polydopamine
PE	polyethylene
PEDOT	poly(3,4-ethylenedioxythiophene)
PEO	poly(ethylene oxide)
PMMA	poly(methyl methacrylate)
PPy	polypyrrole
PSS	poly(styrene sulfonate)
PVA	poly(vinyl alcohol)
PVDF	poly(vinylidene fluoride)
SEI	solid electrolyte interphase
SEM	scanning electron microscopy
SPE	solid polymer electrolyte
TEM	transmission electron microscopy
TFSI	bis(trifluoromethanesulfonyl)imide
XPS	X-ray photoelectron spectroscopy
